# New Thiosemicarbazides and 1,2,4-Triazolethiones Derived from 2-(Ethylsulfanyl) Benzohydrazide as Potent Antioxidants

**DOI:** 10.3390/molecules190811520

**Published:** 2014-08-04

**Authors:** Nafal Nazarbahjat, Nurdiana Nordin, Zanariah Abdullah, Mahmood Ameen Abdulla, Wageeh A. Yehye, Siti Nadiah Abdul Halim, Chin Hui Kee, Azhar Ariffin

**Affiliations:** 1 Department of Chemistry, Faculty of Science, University of Malaya, 50603 Kuala Lumpur, Malaysia; E-Mails: nnazarbahjat@yahoo.com (N.N.); csi_ndiana@um.edu.my (N.N.); zana@um.edu.my (Z.A.); nadiahhalim@um.edu.my (S.N.A.H.); chkee@um.edu.my (C.H.K.); 2 Department of Biomedical Science, Faculty of Medicine, University of Malaya, 50603 Kuala Lumpur, Malaysia; E-Mail: ammeen@um.edu.my; 3 Department of Medical Analysis, College of Health and Medical Technologies, P.O.10047, Baghdad, Iraq; 4 Section for Co-Curriculum Courses, External Faculty Electives & TITAS (SKET), University of Malaya, 50603 Kuala Lumpur, Malaysia; 5 Nanotechnology & Catalysis Research Centre (NANOCAT), University of Malaya, Block 3A, Institute of Postgraduate Studies Building, 50603 Kuala Lumpur, Malaysia; E-Mail: wdabdoub@um.edu.my

**Keywords:** thiosemicarbazide derivatives, 1,2,4-triazoles, thioethers, X-ray structure determination, antioxidant activity

## Abstract

New thiosemicarbazide derivatives **2**–**6** were synthesised by reacting 2-(ethylsulfanyl)benzohydrazide with various aryl isothiocyanates. The cyclisation of compounds **2**–**6** under reflux conditions in a basic medium (aqueous NaOH, 4 N) yielded compounds **7**–**11** that contain a 1,2,4-triazole ring. All of the synthesised compounds were screened for their antioxidant activities. Compounds **2**, **3**, and **7** showed better radical scavenging in a 2,2-diphenyl-1-picrylhydrazyl (DPPH) assay, with IC_50_ values of 1.08, 0.22, and 0.74 µg/mL, respectively, compared to gallic acid (IC_50_, 1.2 µg/mL). Compound **3** also showed superior results in a ferric reducing antioxidant power (FRAP) assay (3054 µM/100 g) compared to those of ascorbic acid (1207 µM/100 g).

## 1. Introduction

The oxidation process represents one of the most important routes for producing reactive oxygen species (ROS). Nitric oxide, hydrogen peroxide, and hydroxyl (OH.) and peroxide (ROO.) radicals are commonly found in foods, drugs, and living systems. These ROS and free radicals may oxidise nucleic acids [1], denature proteins [2], and initiate the peroxidation of lipids [3] and the onset of degenerative diseases [4]. ROS are known to be the main cause of the aging process by their oxidation of cells and tissues [5]. Antioxidants or “oxidation inhibitors” exert their effects by preventing the generation of ROS and retarding the progress of many chronic diseases, including cancer, inflammation and cardiovascular diseases [6]. Many natural as well as synthetic antioxidants are in the market for the treatment of various diseases [7]. Butylated hydroxyanisole (BHA) and butylated hydroxytoluene (BHT) are the most commonly used synthetic antioxidants. Recently, it has been found that these phenolic antioxidants might have carcinogenic potential and even produce toxic effects [8]. These facts have provided the basis for the discovery of new, safer and effective synthetic alternatives. A considerable amount of attention has been devoted to the synthesis of thiosemicarbazide and 1,2,4-triazole derivatives due to their wide range of pharmacological activities, such as their antitumor [9], antibacterial [10], antiproliferative activities [11], and many of them show significant *in vitro* antioxidant activity [12].

A useful strategy for investigating antioxidant activity is through bonding of the antioxidant group with other pharmacophores such as the thioether group. Previous reports have suggested that antioxidants that contain a thioether group are usually more effective than the simple compounds from which they were derived [13]. The design of efficient and economic synthetic routes is often a major factor in new drug discovery. It has been reported that the cyclisation of suitable thiosemicarbazides is an excellent strategy for the synthesis of many heterocyclic derivatives. This includes the formation of 1,3,4-thiadiazole derivatives in acidic media. The same thiosemicarbazides, in the presence of NaOH, underwent cyclisation to yield 1,2,4-triazole thione derivatives [14,15,16].

Working from this hypothesis, a series of 1-[2-(ethylsulfanylphenyl)carbonyl]-4-substituted thiosemicarbazides **2**–**6** and their corresponding cyclised 5-[2-(ethylsulfanyl)phenyl]-4-substituted-2,4-dihydro-3*H*-1,2,4-triazole-3-thiones **7**–**11** were prepared and evaluated for their antioxidant activities using DPPH and FRAP assays.

## 2. Results and Discussion

### 2.1. Synthesis and Spectroscopic Characterization

**Scheme 1:**
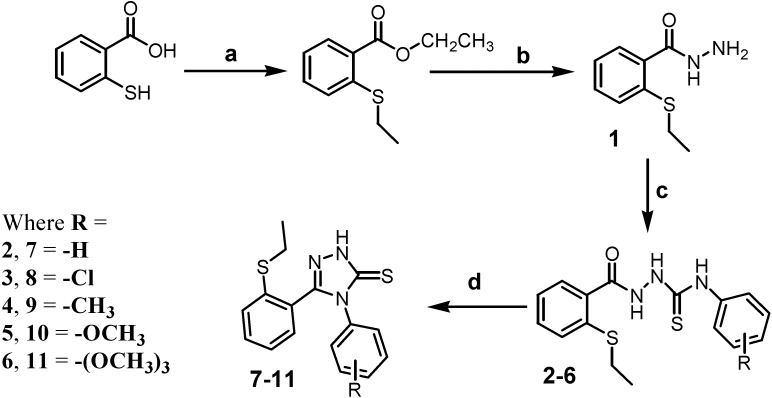
Synthetic pathway of thiosemicarbazides and 1,2,4-triazolethiones.

The preparations of the thiosemicarbazides and 1,2,4-triazoles are outlined in Scheme 1. The 1-[2-(ethylsulfanylphenyl)carbonyl]-4-substitutedthiosemicarbazides **2**–**6** were obtained by reacting 2-(ethylsulfanyl)benzohydrazide (**1**) with arylisothiocyanates in absolute ethanol. Cyclisation of compounds **2**–**6** by aqueous NaOH (4N) yielded the corresponding 5-[2-(ethylsulfanyl)phenyl]-4-substituted-2,4-dihydro-3*H*-1,2,4-triazole-3-thiones **7**–**11**. The structures of these compounds were confirmed by IR, ^1^H-NMR, ^13^C-NMR and mass spectrometry.

**Figure 1:**
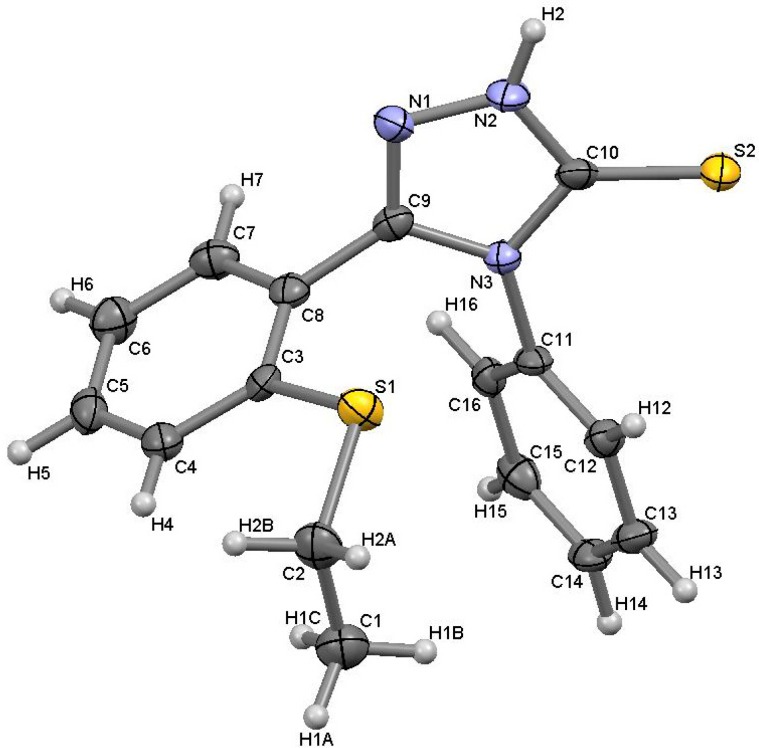
Molecular structure of 5-[2-(ethylsulfanyl)phenyl]-4-phenyl-2,4-dihydro-3*H*-1,2,4-triazole-3-thione (**7**), showing the atomic numbering scheme.

The IR spectra of the thiosemicarbazides **2**–**6** indicated the presence of C=O stretching bands at 1,670–1,640 cm^−1^. The disappearance of the C=O stretching bands and the appearance of strong C=N stretching bands at 1,605–1,590 cm^−1^ is evidence for the ring closure to form the 1,2,4-triazoles **7**–**11**. The formation of the thione tautomer was supported by the presence of absorption maxima at 1,329–1,230 cm^−1^ belonging to the C=S group. The X-ray data of compound **7** (Figure 1) further confirms that the compound exists as the thione tautomer in the solid state.

*Reagents and conditions*: (**a**) K_2_CO_3_, EtBr, Acetone, r.t, 24 h; (**b**) NH_2_NH_2_·H_2_O, EtOH, reflux, 24 h; (**c**) EtOH, arylisothiocyanates, reflux, 1–4 h; (**d**) 4N NaOH, reflux 3 h.

The ^1^H-NMR spectra of compounds **2**–**6** showed that the signal of NHC=O appears at 9.41–9.57 ppm, whereas the NH-Ph and NHC=S peaks appear at 9.71–9.92 and 10.42–10.73 ppm, respectively. The disappearance of the NHC=O and NH-Ph peaks from the ^1^H-NMR spectra and the appearance of a new peak at 14.11–14.21 ppm (N-NHC=S) confirmed the formation of the 1,2,4-triazole-3-thione derivatives **7**–**11**. Figure 1 represents the molecular structure of compound 7. The molecule exists in the solid state in the thione form, with a C=S bond length of 1.683 (3)°.

### 2.2. Antioxidant Activities

#### 2.2.1. DPPH Free Radical Scavenging Activity

**Figure 2:**
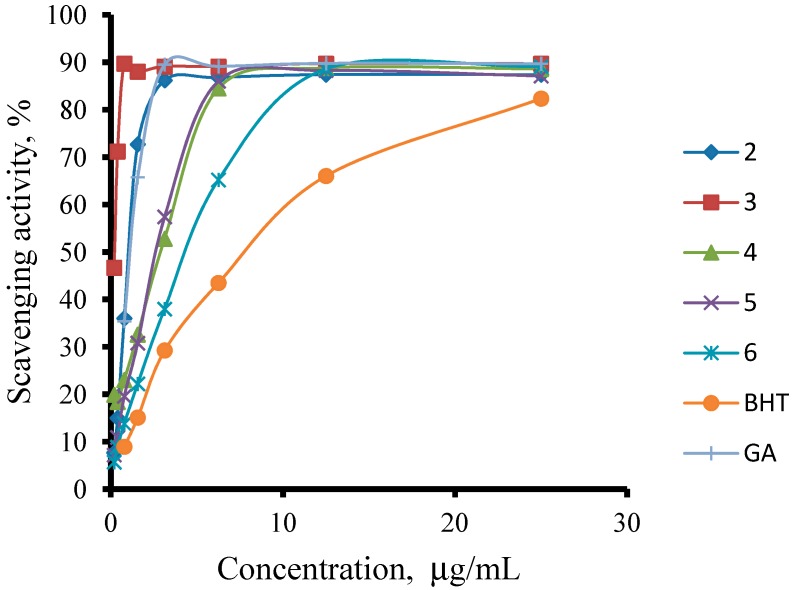
Scavenging activity of compounds **2**–**6** on DPPH radical.

**Figure 3:**
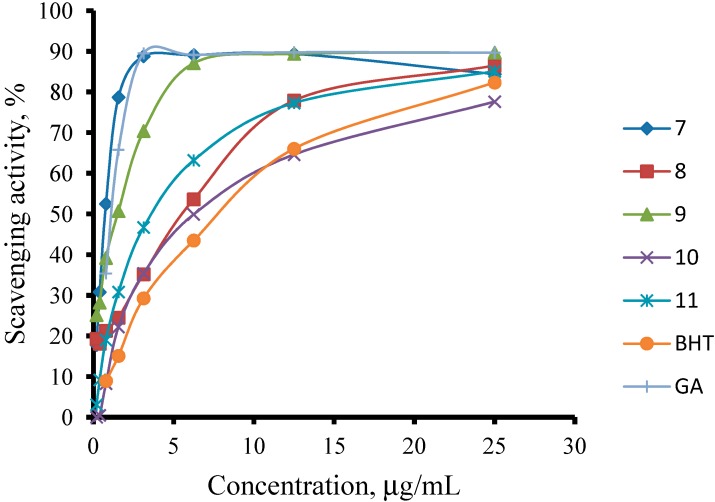
Scavenging activity of compounds **7**–**11** on DPPH radical.

The free radical-scavenging activities of the prepared compounds **2**–**11**, along with those of the reference standards quercetin, BHT, Trolox, rutin, gallic acid and ascorbic acid, were determined using a DPPH assay; the results are shown in Table 1 and Figure 2 and Figure 3.

**Table 1:** Antioxidant activities for compounds **2**–**11**.

Compound	Structure	Yield (%)	DPPH ^a^ (IC_50_^b^ µg/mL)	FRAP ^a^ Values
**2**	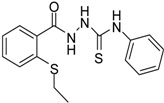	96	1.08 ± 0.02	1193.33 ± 0.05
**3**	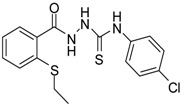	93	0.22 ± 0.01	3054.44 ± 0.15
**4**	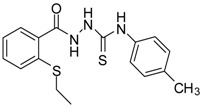	95	2.91 ± 0.05	418.16 ± 0.16
**5**	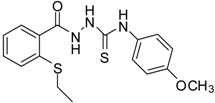	94	2.69 ± 0.21	427.83 ± 0.11
**6**	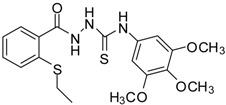	93	4.50 ± 0.01	374.44 ± 0.14
**7**	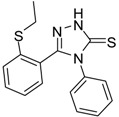	75	0.74 ± 0.15	760.00 ± 0.03
**8**	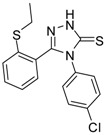	73	5.64 ± 0.03	135.72 ± 0.12
**9**	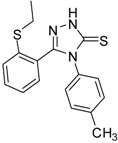	70	1.51 ± 0.08	278.89 ± 0.12
**10**	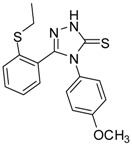	75	6.30 ± 0.13	109.72 ± 0.05
**11**	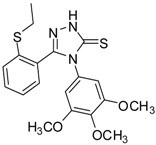	69	3.75 ± 0.01	41.81 ± 0.10
	Quercetin		2.54 ± 0.07	1371.11 ± 0.26
	BHT		18.71 ± 0.01	77.83 ± 0.08
	Trolox		5.35 ± 0.64	987.78 ± 0.14
	Rutin		5.25 ± 0.01	393.89 ± 0.02
	Gallic acid (GA)		1.20 ± 0.13	2957.78 ± 0.05
	Ascorbic acid (AA)		7.52 ± 0.08	1206.67 ± 0.02

^a^ Each value represents mean ± SD; ^b^ IC_50_: 50% effective concentration.

As we can see from the table, all of the thiosemicarbazides possessed greater scavenging effects in the DPPH assay than the standard compounds BHT and ascorbic acid. Compounds **2** and **3** possessed a scavenging effect greater than those for all of the standard compounds, with IC_50_ values of 1.08 ± 0.02 µg/mL and 0.22 ± 0.01 µg/mL, respectively. The corresponding value for the standard antioxidant gallic acid, by contrast, was 1.20 ± 0.13 µg/mL. We also observed that the presence of an electron-withdrawing group (*Cl*) on the phenyl ring of the thiosemicarbazides increased the scavenging ability of the thiosemicarbazide (**3**, IC_50_ = 0.22 ± 0.01 µg/mL) in comparison to that of compound **2** (IC_50_ = 1.08 ± 0.02 µg/mL), which lacks substituents. By contrast, the presence of an electron-donating group (*OMe*) decreased the scavenging ability (**6**, IC_50_ = 4.50 ± 0.01 µg/mL).

However, with the exception of compounds **7** and **9**, the cyclisation of the thiosemicarbazides to their corresponding 1,2,4-triazole thione derivatives did not improve the radical-scavenging activity relative to the uncyclised compounds. Only compounds **7** and **9** showed an improvement in the radical scavenging activity, with IC_50_ values of 0.74 ± 0.15 µg/mL and 1.51 ± 0.08 µg/mL, respectively, compared to those of their corresponding thiosemicarbazides **2** and **4** (IC_50_ 1.08 ± 0.02 and 2.91 ± 0.05 µg/mL, respectively). Compound **3** possessed better scavenging properties than its cyclised product, **8**. Unlike for the thiosemicarbazides, there was no correlation between the substituent group on the phenyl ring and the scavenging ability of the 1,2,4-triazole thione compounds.

**Scheme 2:**
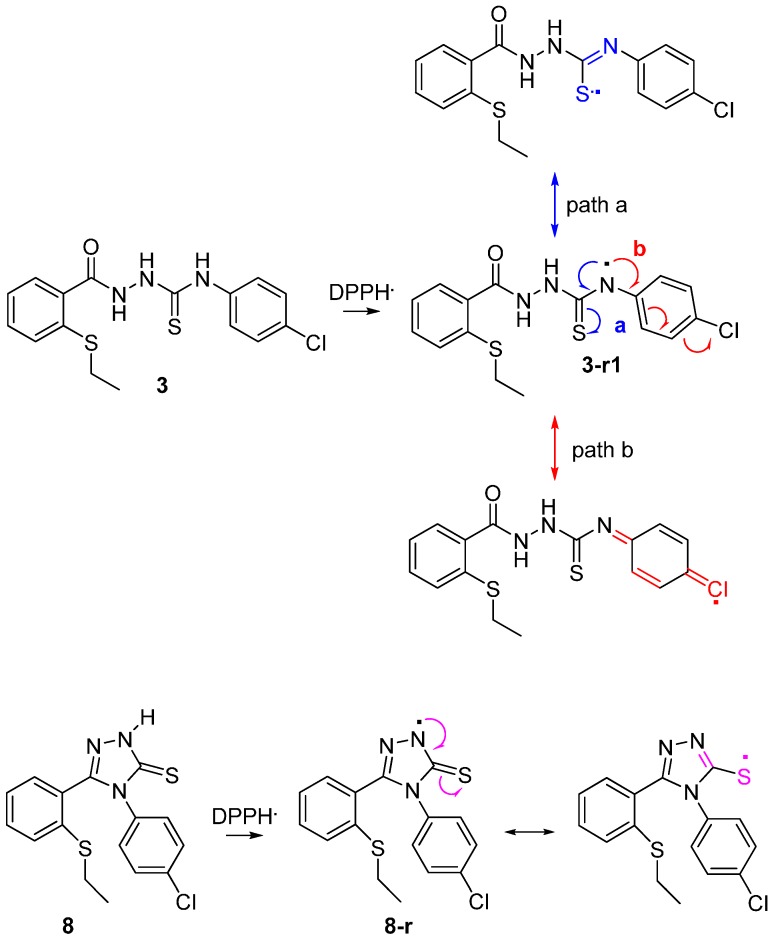
Proposed mechanism to account for the fact that thiosemicarbazide **3** has superior radical-scavenging effects compared to **8**.

This may be explained by the mechanism shown on Scheme 2. The electron-withdrawing substituent on the phenyl ring has an influence on the radical-scavenging effects of the thiosemicarbazide **2** by delocalisation of the nitrogen centred radical. For the 1,2,4-triazole thione derivatives, delocalisation across the phenyl ring is not an option. The chlorine atom is a strongly electron-withdrawing atom by induction, whereas the –OMe group is a strongly electron-donating group by resonance.

#### 2.2.2. Ferric ions (Fe+3) Reducing Antioxidant Power (FRAP) Assay

**Figure 4:**
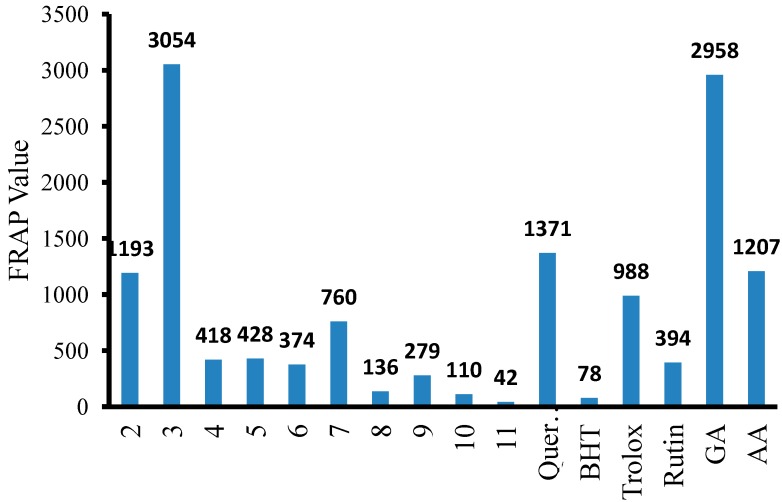
FRAP Value for compound **2**–**11** and reference standard.

The ferric reducing antioxidant power (FRAP) assay relies on the ability of an antioxidant to reduce the yellow ferric tripyridyltriazine complex (Fe(III)-TPTZ) to the blue ferrous complex (Fe(II)-TPTZ) by the action of electron-donating antioxidants. The absorbance of the coloured complex (Fe(II)-TPTZ) is monitored spectrophotometrically at 593 nm [17]. All compounds exhibited reducing power except for compound **11** (substituted with three –OMe groups), which showed a very poor FRAP value (41.81 ± 0.11) when compared to the reference standards, as shown in Table 1 and Figure 4.

The phenyl derivative **2** exhibited a good FRAP value (1193.33 ± 0.05) when compared to that of ascorbic acid (1206.67 ± 0.02). The result indicated that the *p*-chlorophenyl derivative compound **3** had the highest FRAP value (3054.44 ± 0.01), which is above the value for gallic acid (2957.78 ± 0.05). Consequently, compounds **4**, **5**, and **7** displayed FRAP values (418.16 ± 0.19, 427.83 ± 0.11 and 760.00 ± 0.03, respectively) higher than that of rutin (393.89 ± 0.02) but lower than that of Trolox (987.78 ± 0.14).

In addition, compounds **6**, **8**, **9** and **10** possessed FRAP reducing power values (374.4 ± 0.14, 135.7 ± 0.19, 278.8 ± 0.15 and 109.7 ± 0.05, respectively) higher only than that of BHT (77.83 ± 0.08). The results obtained from the FRAP assay clearly indicated that all of the thiosemicarbazides showed better FRAP reducing power than the related 1,2,4-triazoles.

As in the case of the DPPH assay, the FRAP assay of the thiosemicarbazides also shows a similar pattern. The presence of an electron withdrawing group (*Cl*) increase the reducing power of the thiosemicarbazides (**3**, 3054.44 ± 0.01) in comparison to that of compound **2** (1206.67 ± 0.02), which lacks substituents. By contrast, the presence of an electron-donating group (*OMe*) decreased the reducing power ability (**6**, 374.4 ± 0.14). As for the 1,2,4-triazoles, there was no direct correlation between the substituent on the phenyl ring and the reducing power ability of the compounds.

The DPPH assay involves both hydrogen atom transfer (HAT) and electron transfer *(*ET) mechanisms, whereas FRAP assay involves ET mechanism [18]. HAT-based methods measure the classical ability of an antioxidant to scavenge free radicals by hydrogen donation to form stable compounds. ET-based methods detect the ability of a potential antioxidant to transfer one electron to reduce any compound, including metals, carbonyls, and radicals [19]. In the present study, we proposed that the DPPH assay involve the HAT mechanism as described earlier. However we are unable to provide a mechanism that could explain how the substituent on the phenyl ring could effects on the reducing power ability of the compounds.

#### 2.2.3 Quantum Calculation of the Antioxidant Activities of Compounds **3** and **8**

**Figure 5:**
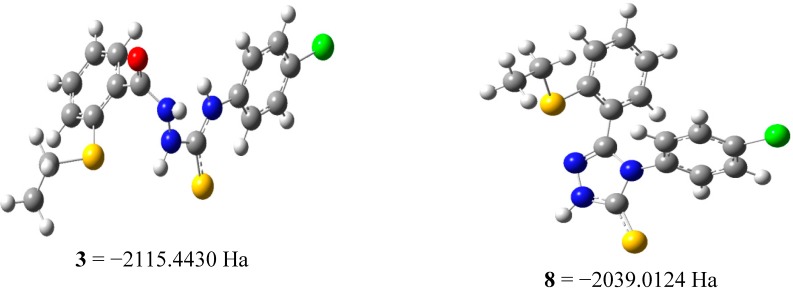
The uB3LYP/6-31G (d,p) optimized geometries of the compounds **3** and **8**.

A computational analysis of the relative radical stabilities and bond-dissociation enthalpies (BDH_298_) based on DFT (uB3LYP/6-31G (d, p)) calculations enable us to rationalise the experimental results [20,21]. By calculating the spin density on the radical intermediate, we could predict which intermediate would be expected to be more stable. All calculations were performed using Gaussian 09W based on DFT [20,21]. Figure 5 shows the optimized geometry of the compound’s radical. It was our intention to employ uB3LYP/6-31G (d, p) level of theory to perform the most reliable optimization at the geometrical parameters of these compounds.

From the data, the energy level of compound **8** at the neutral state is higher than the energy of compound **3**. This indicates that compound **3** will be able to form a stable radical.

The hydrogen atom of –NH in the compounds above is obstructed by radical to form the N radical, -N. The thiosalicyclic rings are connected with amine group to form a conjugative system in the compounds. This will be beneficial to the N atom when the compounds form radicals. Therefore, the corresponding radicals formed is relatively stable if the spin density on N atom in the compounds is low [22].

**Figure 6:**
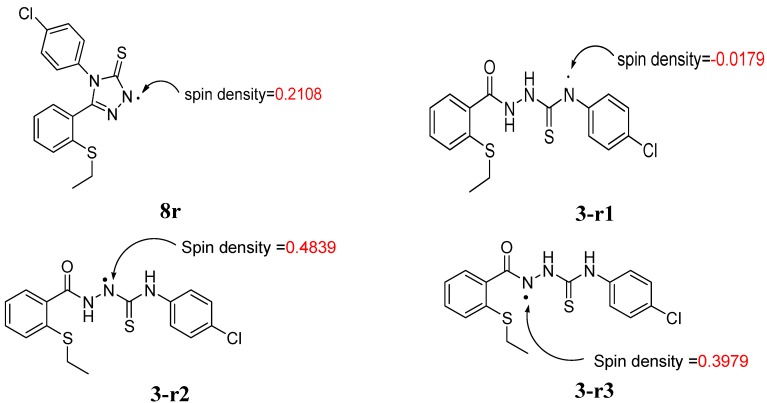
Spin density in the -N radical of compound **8r** and compound **3** radicals at uB3LYP/6-31G (d,p).

The result are represented in Figure 6. The spin density values were able to help us in understanding the difference of the antioxidant activities among the compounds.

The sequence η**_3-r1_** < η**_8_** < η**_3_**_-**r3**_ < η**_3-r2_** indicates that antioxidant ability of compound **3** is higher than that of compound **8**. This can be explained by the low spin density on -N radical in compound **3-r1** [22]. The single electron in compound **3** can be dispersed to the benzene ring [22]. The low antioxidant activity of compound **8** may also be due to the less resonance radical in the system. A relative low spin density on N atom in compound **3r1** implicates that the opened chain exhibit a much more electron rich than the closed ring in compound **8** [22].

**Figure 7:**
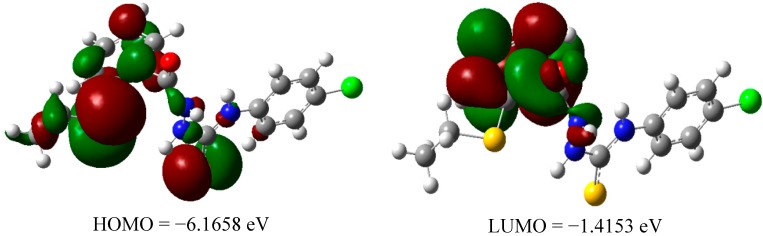
The HOMO LUMO electron distribution of compound **3**.

**Figure 8:**
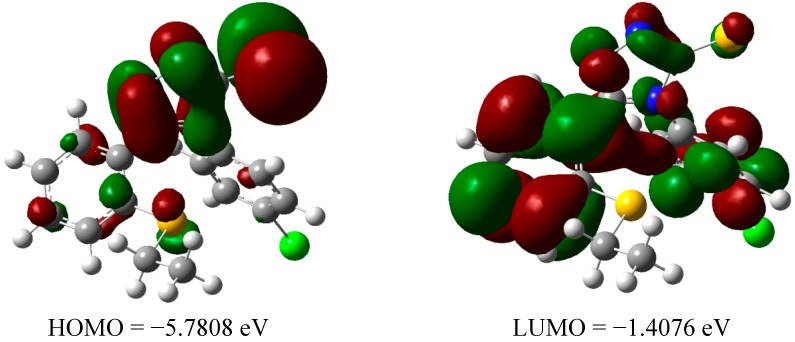
The HOMO LUMO electron distribution of compound **8**.

The equilibrium geometries of neutral **3** and **8** in the form of orbital composition of their highest occupied molecular orbital (HOMO) and the lowest unoccupied molecular orbital (LUMO) are illustrated in Figure 7 and Figure 8, respectively. A few notable differences are found in the computed electronic structures of the compounds **3** and **8**. The charge density is highly delocalized over one part of the molecules. The charge density concentrated on the amine group (for compound **3**) and the triazole ring (for compound **8**). A big difference is noticed at LUMO composition between these two compounds. The electron density distribution focused on the whole molecule for compound **8** whereas for compound **3** the electron distribution only concentrated on the aromatic system. As far as electronic structures of the radical species of both compounds are concerned, it is obvious that the charge density is much more delocalized when the hydrogen atom abstraction took place. The lower energy of the LUMO in compound **3** (a more powerful inhibitors of mutagenesis) is an indication that the compound can behave as soft electrophiles. On the other hand, higher value of HOMO in compound **3** indicates the ability to donate electron is higher when compared to compound **8**.

Because the DPPH assay involves the hydrogen atom transfer (HAT) mechanism, calculations of BDH_298_ could further support the experimental data. Using this method, we calculated the BDH_298_ for compounds **3**-**r1**, **3**-**r2**, **3**-**r3** and **8**-**r**, and the results are summarised in Table 2 [23]

Table 2 showed the value of BDH for compounds **3** and **8**. The DFT results appeared to be quite realistic for phenol compounds. In this case, the BDH values for the NH sites on the open chained system, **3r1** (414.8133 kcal/mol) is lower than the closed chain (425.7250 kcal/mol). This clearly confirms that H-atom transfer (HAT) from the open chain system of compound **3r1** is easier than the closed system of compound **8**. The results indicate that the reactivity of the open chain system for compound **3r1**, is higher than the reactivity for closed chain system for compound **8**. Thus, compound **3** has the capacity to impact significat activity by acting as a hydrogen atom donor and also by enabling the formation of a relatively stable radical when formed through electron delocalization.

**Table 2:** Optimized geometries and BDH_298_ values of radicals derived from **3** and **8** for gas phase calculations.

Compd	ΔH^rxn^ (kcal/mol)	ΔG^rxn^ (kcal/mol)	BDH_298_ (kcal/mol)
**3r1**	7.4370	8.9157	414.8133
**3r2**	7.4546	8.9520	426.4630
**3r3**	7.4470	7.3645	436.3319
**8r**	8.4776	7.9895	425.7250

**Figure 9:**
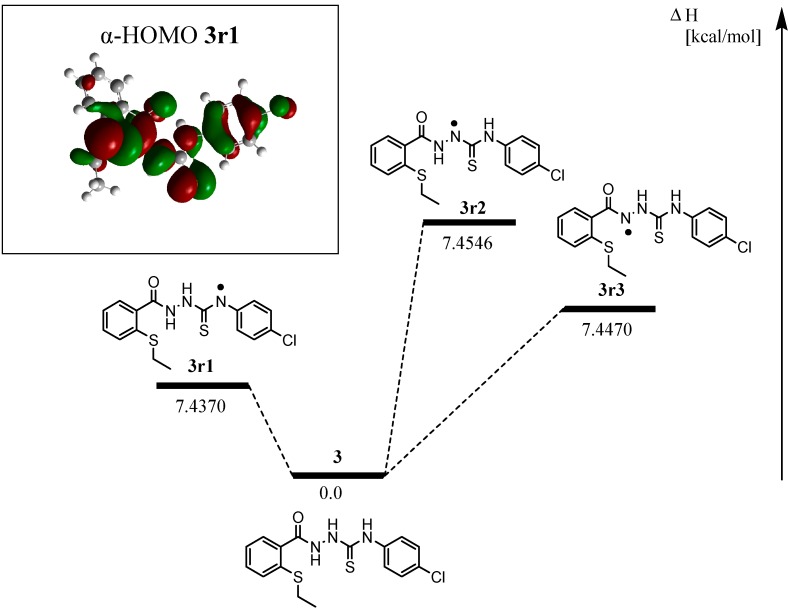
Bond-dissociation enthalpies for three radicals in compound **3**. Stabilization of the radical in **3r1** is more longer by the adjacent sulphur and hyperconjugation lead to a rather BDH of 7.4370 kcal/mol (uB3LYP/6-31 G (d,p)).

Compound **3r1** is known to be stable as they benefit from inductive effects as well as from orbital interactions of the p-type lone pair of sulphur atom with the half-filled p-orbital of the mainly sp^2^ hybridized radicals [23]. The picture of the SOMO and the mapped out spin density of **3r1** illustrate the final effect (Figure 9). On top of that, the position of the radical helped to stabilize the radical further, mainly by hyperconjugation. Addition of –Cl as a withdrawing electron group may also improve the stability of the conjugation.

## 3. Experimental

### 3.1. Chemistry

#### 3.1.1. General Information

All of the chemicals and solvents were obtained from Sigma-Aldrich (Petaling Jaya, Selangor, Malaysia) and used without further purification. The melting points were determined by using a MEL-TEMP II apparatus and were uncorrected. The IR spectra were recorded from 4,000 to 400 cm^−1^ using a Perkin Elmer 400 Fourier transform infrared (FTIR) spectrometer. The ^1^H-NMR and ^13^C-NMR spectra were recorded on a Bruker-AVN III 400-MHz instrument using CDCl_3_ and DMSO-*d*_6_ as the solvents and TMS as an internal standard. The mass spectra were recorded on a Finnegan TSQ7000 for HREI/MS.

#### 3.1.2. General Procedure for the Synthesis of 1-[(2-Ethylsulphanylphenyl)carbonyl]-4-substituted Thiosemicarbazide Derivatives **2**–**6**

A solution (1 mmol) of 2-(ethylsulfanyl)benzohydrazide (**1**) and an appropriate amount of isothiocyanate in anhydrous ethanol (15 mL) was heated under reflux for 1–4 h. The solution was cooled, and the solid formed was filtered off, washed with diethyl ether, dried, and recrystallised from EtOH.

*1-[(2-Ethylsulfanylphenyl)carbonyl]-4-phenyl thiosemicarbazide* (**2**). Colourless solid. Yield 96%, m.p. 120 °C, IR (KBr) (*v*, cm^−1^): 3260 (N-H st), 2972 (C-H st), 1643 (C=O st), 1598 (C=N st), 1239 (C=S st). ^1^H-NMR (DMSO-*d*_6_) δ ppm: 1.2 (t, 3H, CH_3_), 2.95 (q, 2H, -CH_2_), 7.18 (m, 1H, Ar-H) 7.29 (m, 1H, Ar-H), 7.37 (t, *J* = 7.8, 2H, Ar-H), 7.51 (m, 4H, Ar-H), 7.74 (d, *J* = 7.5, 1H, Ar-H), 9.53 (br s, 1H, NHPh), 9.84 (br s, 1H, NHCS), 10.42 (s, 1H, NHCO). ^13^C-NMR (DMSO-*d*_6_) δ ppm: 13.64, 26.56, 124.93, 128.23, 128.63, 130.82, 133.93, 136.24, 138.97, 167.02, 180.97. HREIMS *m/z* 331.0816 [M^+^] (calc. for C_16_H_17_N_3_O_1_S_2_ 331.0813).

*1-[(2-Ethylsulfanylphenyl)carbonyl]-4-(4-chlorophenyl)thiosemicarbazide* (**3**). Colourless solid. Yield 93%, m.p. 150 °C, IR (KBr) (*v*, cm^−1^): 3179 (N-H st), 1666 (C=O st), 1587 (C=N st), 1223 (C=S st). ^1^H-NMR (DMSO-*d*_6_) δ ppm: 1.21 (t, 3H, CH_3_), 2.95 (q, 2H, -CH_2_), 7.28 (m, 1H, Ar-H), 7.43 (m, 4H, Ar-H), 7.55 (d, *J* = 7.8, 2H, Ar-H), 7.75 (d, *J* = 7.7, 1H, Ar-H), 9.57 (br s, 1H, NH-Ph), 9.92 (br s, 1H, NHCS), 10.41 (s, 1H, NHCO). ^13^C-NMR (DMSO-*d*_6_) δ ppm: 13.63, 26.37, 124.76, 126.99, 127.85, 128.107, 128.70, 130.88, 133.50, 136.56, 138.03, 166.99, 180.86. HREIMS *m/z* 365.0419 [M^+^] (calc. for C_16_H_16_N_3_O_1_S_2_ 365.0423).

*1-[(2-Ethylsulfanylphenyl)carbonyl]-4-(4-methylphenyl)thiosemicarbazide* (**4**). Colourless solid. Yield 95%, m.p. 120 °C, IR (KBr) (*v*, cm^−1^): 3240 (NH st), 1641 (C=O st), 1615 (C=N st), 1237 (C=S st). ^1^H-NMR (DMSO-*d*_6_) δ ppm: 1.19 (t, 3H, CH_3_), 2.3 (s, 3H, -CH_3_), 2.94 (q, 2H, -CH_2_), 7.16 (m, *J* = 8.2, 2H, Ar-H), 7.28 (dt, *J* = 8, 4.1, 1H, Ar-H), 7.37 (m, *J* = 8, 2H, Ar-H), 7.48 (d, *J* = 3.9, 2H, Ar-H), 7.72 (d, *J* = 7.5, 1H, Ar-H), 9.44 (br s, 1H, NHPh), 9.76 (br s, 1H, NHCS), 10.38 (s. 1H, NHCO). ^13^C-NMR (DMSO-*d*_6_) δ ppm: 13.65, 20.50, 26.65, 125.02, 125.24, 128.18, 128.158, 128.71, 130.82, 134.05, 134.27, 136.09, 136.32, 138.36, 167.15, 181.02. HREIMS *m/z* 345.0956 [M^+^] (calc. for C_17_H_19_N_3_O_1_S_2_ 345.0970).

*1-[(2-ethylsulfanylphenyl)carbonyl]-4-(4-methoxyphenyl)thiosemicarbazide* (**5**). Colourless solid. Yield 94%, m.p. 172–174 °C, IR (KBr) (*v*, cm^−1^): 3267 (NH st), 1667 (C=O st), 1607 (C=N st), 1354 (C=S st). ^1^H-NMR (DMSO-*d*_6_) δ ppm: 1.19 (t, 3H, CH_3_), 2.94 (q, 2H, -CH_2_), 3.76 (s, 3H, -OCH_3_), 6.93 (m, 2H, Ar-H), 7.29 (m, 1H, Ar-H), 7.35 (m, *J* = 8.5, 2H, Ar-H), 7.48 (d, *J* = 4, 2H, Ar-H), 7.73 (d, *J* = 7.5, 1H, Ar-H), 9.41 (br s, 1H, NH-Ph), 9.71 (br s, 1H, NHCS), 10.37 (s, 1H, NHCO). ^13^C-NMR (DMSO-*d*_6_) δ ppm: 13.65, 26.61, 55.22, 113.43, 124.96, 126.79, 128.13, 128.64, 130.81, 131.74, 134.0, 136.15, 156.79, 167.05, 181.16. HREIMS *m/z* 361.0907 [M^+^] (calc. for C_17_H_19_N_3_O_2_S_2_ 361.0919).

*1-[(2-Ethylsulfanylphenyl)carbonyl]-4-(3,4,5-trimethoxyphenyl)thiosemicarbazide* (**6**). Colourless solid. Yield 93%, m.p. 156–158 °C, IR (KBr) (*v*, cm^−1^): 3545, 3355, 3283 (NH st), 2969 (C-H st), 1651 (C=O st), 1594 (C=N st), 1339 (C=S st). ^1^H-NMR (DMSO-*d*_6_) δ ppm: 1.21 (t, 3H, CH_3_), 2.95 (q, 2H, -CH_2_), 3.66-3.76 (s, 9H, -OCH_3_), 6.91 (s, 2H, Ar-H), 7.28 (dt, *J* = 7.5, 1H, Ar-H), 7.48 (br s, 2H, Ar-H), 7.73 (d, *J* = 7.4, 1H, Ar-H), 9.41 (br.s., 1H, NHPh), 9.79 (br s; 1H, NHCS), 10.37 (s., 1H, NHCO). ^13^C-NMR (DMSO-*d*_6_) δ ppm: 13.68, 26.61, 55.85, 60.07, 102.25, 124.90, 128.05, 128.66, 130.84, 133.88, 134.67, 134.79, 136.30, 152.33, 166.96, 180.47. HREIMS *m/z* 421.1114 [M^+^] (calc. for C_19_H_23_N_3_O_4_S_2_ 421.1130).

#### 3.1.3. General Procedure for the Synthesis of 5-[2-(Ethylsulfanyl)phenyl]-4-substituted-2,4-dihydro-3*H*-1,2,4-triazole-3-thiones **7**–**11**

Appropriate substituted thiosemicarbazides **2**–**6** (0.01 mol) were dissolved in 4 N aqueous sodium hydroxide (15 mL) and refluxed for 3 h. After cooling, the mixture was neutralised with 3 M hydrochloric acid. The precipitate formed was filtered and washed several times with distilled water.

*5-[2-(Ethylsulfanyl)phenyl]-4-phenyl-2,4-dihydro-3H-1,2,4-triazole-3-thione* (**7**). Colourless solid. Yield 75%, m.p. 180 °C, IR (KBr) (*v*, cm^−1^): 3260, 3031 (NH st), 2970 (C-H st), 1634 (C=N st), 1596 (C=C st), 1326 (C=S st). ^1^H-NMR (DMSO-*d*_6_) δ ppm: 1.1 (t, 3H, CH_3_), 2.86 (q, 2H, -CH_2_), 7.19 (m, 1H, Ar-H), 7.29 (m, 2H, Ar-H), 7.37 (m, 7H, Ar-H), 7.46 (dd, *J* = 7.6, 1H, Ar-H), 14.14 (s, 1H, NH),.^13^C-NMR (DMSO-*d*_6_) δ ppm: 13.63, 26.52, 125.31, 126.06, 127.94, 128.07, 128.66, 128.97, 131.19, 131.65, 133.66, 137.65, 149.82, 167.64. HREIMS *m/z* 313.0712 [M ^+^] (calc. for C_16_H_15_N_3_S_2_ 313.0707).

*4-(4-Chlorophenyl)-5-[2-(ethylsulfanyl)phenyl]-2,4-dihydro-3H-1,2,4-triazole-3-thione* (**8**). Colourless solid. Yield 73%, m.p. 230–232 °C, IR (KBr) (*v*, cm^−1^): 3194 (NH st), 1557 (C=C st), 1245 (C=S st). ^1^H-NMR (DMSO-*d*_6_) δ ppm: 1.07 (t, 3H, CH_3_), 2.83 (q, 2H, -CH_2_), 7.21 (td, *J* = 7.6, 1H, Ar-H), 7.29 (m, 2H, Ar-H), 7.37 (d, *J* = 7.5, 1H, Ar-H), 7.43 (m, 4H, Ar-H), 14.23 (br.s., 1H, NH). ^13^C-NMR (DMSO-*d*_6_) δ ppm: 14.01, 26.90, 125.96, 128.33, 129.33, 130.41, 132.01, 132.10, 132.84, 134.31, 138.01, 150.29, 167.95. HREIMS *m/z* 347.0316 [M^+^] (calc. for C_16_H_14_N_3_S_2_Cl 347.0318).

*5-[2-(Ethylsulfanyl)phenyl]-4-(4-methylphenyl)-2,4-dihydro-3H-1,2,4-triazole-3-thione* (**9**). Colourless solid. Yield 70%, m.p.220 °C, IR (KBr) (*v*, cm^−1^): 3091 (NH st), 2968 (C-H st), 1642 (C=N st), 1599 (C=C st), 1329 (C=S st). ^1^H-NMR (DMSO-*d*_6_) δ ppm: 1.12 (t, 3H, CH_3_), 2.26 (s, 3H, -CH_3_), 2.87 (q, 2H, CH_2_), 7.16 (s, 4H, Ar-H), 7.19 (td, *J* = 7.3, 1.6, 1H, Ar-H), 7.41 (m, 3H, Ar-H), 14.11 (s, 1H, NH).^13^C-NMR (DMSO-*d*_6_) δ ppm: 14.13, 21.09, 27.04, 125.82, 126.65, 128.29, 128.45, 129.68, 131.59, 131.69, 132.09, 138.17, 139.09, 150.40, 168.20. HREIMS *m/z* 327.0869 [M^+^] (calc. for C_17_H_17_N_3_S_2_ 327.0864).

*5-[2-(Ethylsulfanyl)phenyl]-4-(4-methoxyphenyl)-2,4-dihydro-3H-1,2,4-triazole-3-thione* (**10**). Colourless solid. Yield 75%, m.p. 184 °C, IR (KBr) (*v*, cm^−1^): 3328 (NH st), 1607 (C=N st), 1593 (C=C st), 1346 (C=S st). ^1^H-NMR (DMSO-*d*_6_) δ ppm: 1.11 (t, 3H, CH_3_), 2.87 (q, 2H, -CH_2_), 3.71 (s, 3H, -OCH_3_), 6.9 (m, 2H, Ar-H), 7.19 (m, 3H, Ar-H), 7.42 (m, 3H, Ar-H) 14.12 (s, 1H, NH).^13^C-NMR (DMSO-*d*_6_) δ ppm: 13.63, 26.49, 55.27, 113.83, 125.32, 126.09, 126.17, 127.89, 129.28, 131.21, 131.57, 137.63, 150.02, 159.22, 167.80. HREIMS *m/z* 343.0811 [M^+^] (calc. for C_17_H_17_N_3_OS_2_ 343.0813).

*5-[2-(Ethylsulfanyl)phenyl]-4-(3,4,5-trimethoxyphenyl)-2,4-dihydro-3H-1,2,4-triazole-3-thione* (**11**). Colourless solid. Yield 69%, m.p. 180–182 °C, IR (KBr) (*v*, cm^−1^): 3329 (NH st), 1555 (C=C st), 1251 (C=S st). ^1^H-NMR (DMSO-*d*_6_) δ ppm: 1.11 (t, 3H, CH_3_), 2.89 (q, 2H, -CH_2_), 3.34-3.63 (s, 9H, -OCH_3_), 6.65 (s, 2H, Ar-H), 7.23 (t, *J* = 7.4, 1H, Ar-H), 7.44 (m, 2H, Ar-H), 7.52 (d, *J* = 7.5, 1H, Ar-H), 14.13 (br.s., 1H, NH). ^13^C-NMR (DMSO-*d*_6_) δ ppm: 13.66, 26.43, 55.98, 59.99, 105.98, 125.21, 126.13, 127.73, 129.03, 131.23, 131.71, 137.41, 137.82, 149.89, 152.34, 167.44. HREIMS *m/z* 403.1030 [M^+^] (calc. for C_19_H_21_N_3_O_3_S_2_ 403.1024).

### 3.2. Single Crystal X-ray Structure Determination

Diffraction data were obtained using a Bruker SMART Apex II CCD area-detector diffractometer equipped with graphite-monochromated Mo Kα radiation. The orientation matrix, unit cell refinement and data reduction were all handled by the *CryAlis*^PRO^ software [24]. The structures were solved using the direct method in the program SHELXS-97 and were refined by the full matrix least-squares method on *F^2^* with SHELXL-97 [25]. Drawing of the molecule was performed with *X-Seed* [26]. CCDC 938977 contains the supplementary crystallographic data for **7**. Crystal data may be obtained on request from the authors or free of charge via http://www.ccdc.cam.ac.uk/conts/ retrieving.html and also from the Cambridge Crystallographic Data Centre, 12 Union Road, Cambridge CB2 1EZ, UK; fax: (+44)-1223-336-033; or e-mail: deposit@ccdc.cam.ac.uk.

### 3.3. Pharmacological Assays

#### 3.3.1. DPPH Free Radical Scavenging Activity

The determination of the radical-scavenging activity of the compounds was performed as reported [27]. The 100 µM solution of DPPH (195 µL) in 96% ethanol was added to the tested sample solution (5 µL) in ethanol and mixed in a 96-well plate. Test compounds were allowed to react with the stable free radical 1,1-diphenyl-2-picrylhydrazyl radical (DPPH) for 3 h at 37 °C. After incubation, a decrease in absorption was measured at 515 nm using a spectrophotometer. The percent radical-scavenging activity was calculated using the following equation:

DPPH radical scavenged (%) = [OD Blank − OD Sample ]/[OD Blank] × 100%

where the OD blank is the absorbance of the control DPPH solution, and the OD sample is the tested compound absorbance. The IC_50_ (compound concentration required to reduce the absorbance of the DPPH control solution by 50%) value was then calculated.

#### 3.3.2. Ferric Reducing Antioxidant Power (FRAP) Assay

The reducing capacities of the prepared compounds were measured by the method of Benzie and Strain with a modification [17]. First, 10 mL of acetate buffer (300 mM) was adjusted to pH 3.6 by mixing with 3.1 g CH_3_COONa∙3H_2_O and 16 mL glacial acetic acid. Next, a TPTZ solution was prepared by dissolving 10 mM TPTZ in 40 mM HCl. Then, 1 mL of the (2,4,6-tripyridyl-s-triazine) TPTZ solution was mixed with the FRAP solution, and 1 mL of ferric chloride hexahydrate (20 mM) in a distilled water. The FRAP solution was warmed to 37 °C, the tested compound was added to it, and the mixture was left to react in the dark. The absorbance was monitored spectrophotometrically at 593 nm. The results were expressed in µM ferrous/g dry mass and compared to those for the reference compounds.

#### 3.3.3. Computational Studies

All computations were performed using the GAUSSIAN 09W software package [28]. ChemSketch and GaussView visualisation were used to present the images in the figures. The optimisation structures were calculated by the B3LYP/6-311G (d, p) method [29,30]. Our calculation includes the frontier orbital HOMO and LUMO energies, BDE on each NH site, and the spin-density distribution for the radicals formed after H-removal. The conformer with the lowest electronic energy was used for calculation. The haemolytic BDE values were calculated by the following relationship, using the standard-state enthalpies at 1 atm and 298.15K:

BDE = H_radical_ + H_H_ − H_molecule_ (1)

where H_radical_ is the total enthalpy of the free radical, H_H_ is the gas-phase total enthalpy of the hydrogen atom, and H_molecule_ is the total enthalpy of the parent molecule.

## 4. Conclusions

A new series of thiosemicarbazides **2**–**6** and 1,2,4-triazole derivatives **7**–**11** were prepared. The antioxidant activity of these compounds was evaluated using DPPH and FRAP assays, two methods that act through different mechanisms. A comparison of the results obtained from both assays revealed that all of the thiosemicarbazide derivatives showed better antioxidant activity than the 1,2,4-triazole derivatives. Compounds **2**, **3** and **7** showed excellent antioxidant activities that were higher than that of the standard gallic acid. Based on our findings, further studies would be of value, especially for the development of newly synthesised antioxidants.

## Supplementary Materials

Supplementary materials can be accessed at: http://www.mdpi.com/1420-3049/19/8/11520/s1.

## Supplementary Files

Supplementary File 1
