# Supplementary material for: New Thiosemicarbazides and 1,2,4-Triazolethiones Derived from 2-(Ethylsulfanyl) Benzohydrazide as Potent Antioxidants
Source: Molecules. 2014 Aug 4;19(8):11520–37. doi: 10.3390/molecules190811520 (PMC6271535; doi:10.3390/molecules190811520)
Supplement: Supplementary File 1 [file molecules-19-11520-s001.pdf]

Supporting Information

Figure S1. ^{1}H spectrum of compound **2** in DMSO-d_6.

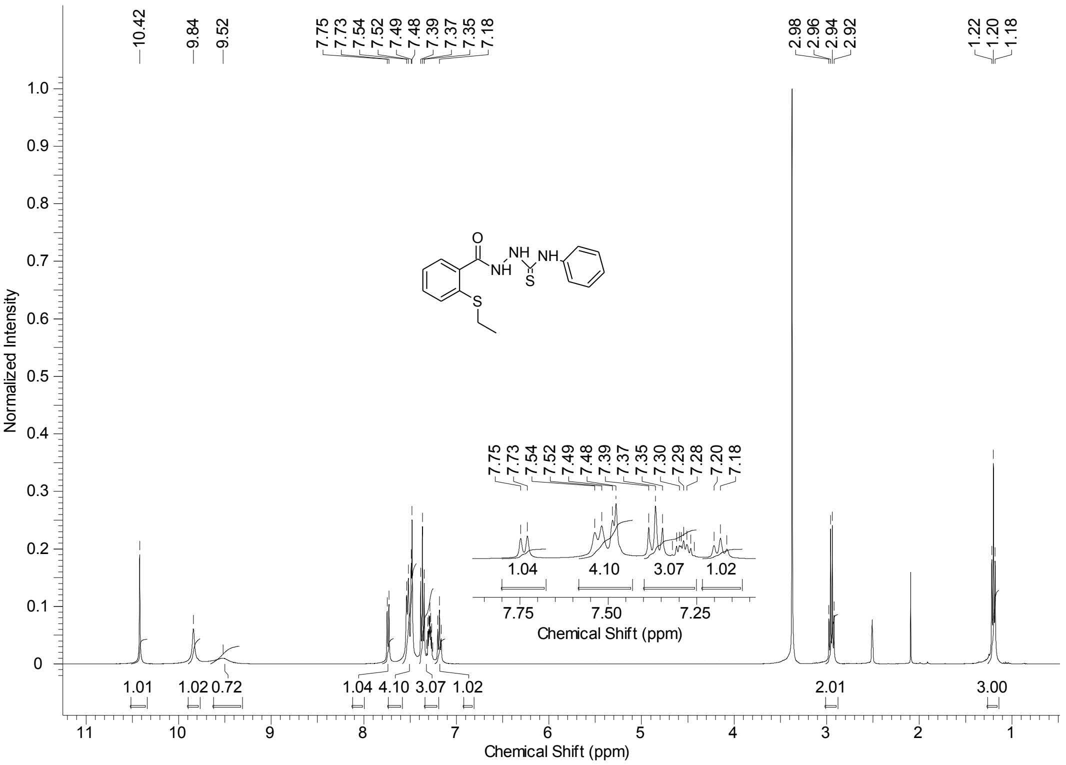

Figure S2. ^{13}C spectrum of compound **2** in DMSO-d_6.

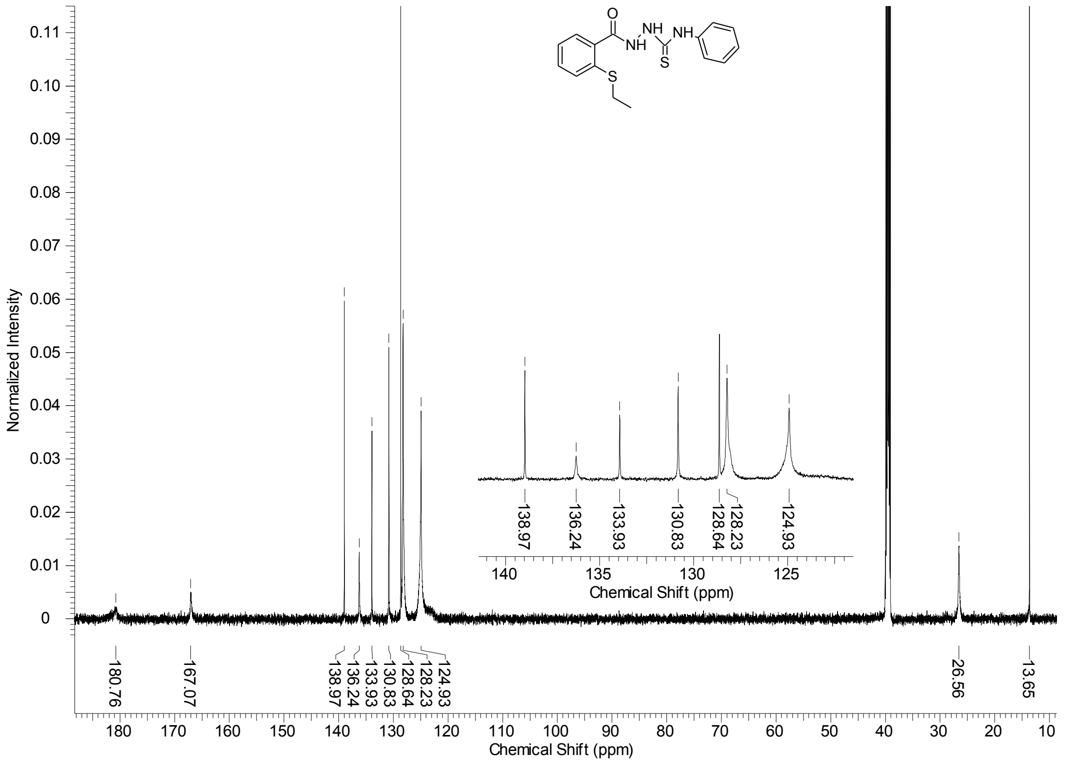

Figure S3. ^{1}H spectrum of compound **7** in DMSO-d_6.

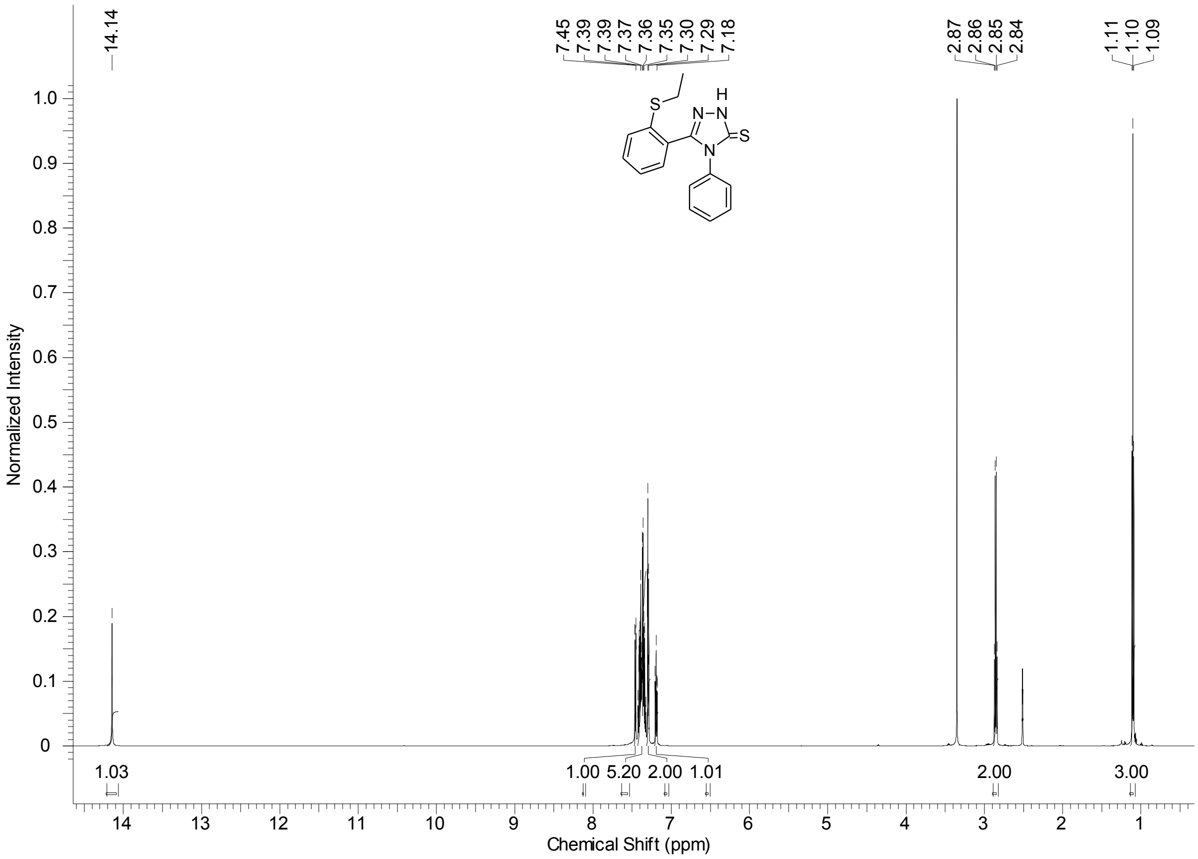

Figure S4. ^{13}C spectrum of compound **7** in DMSO-d_6.

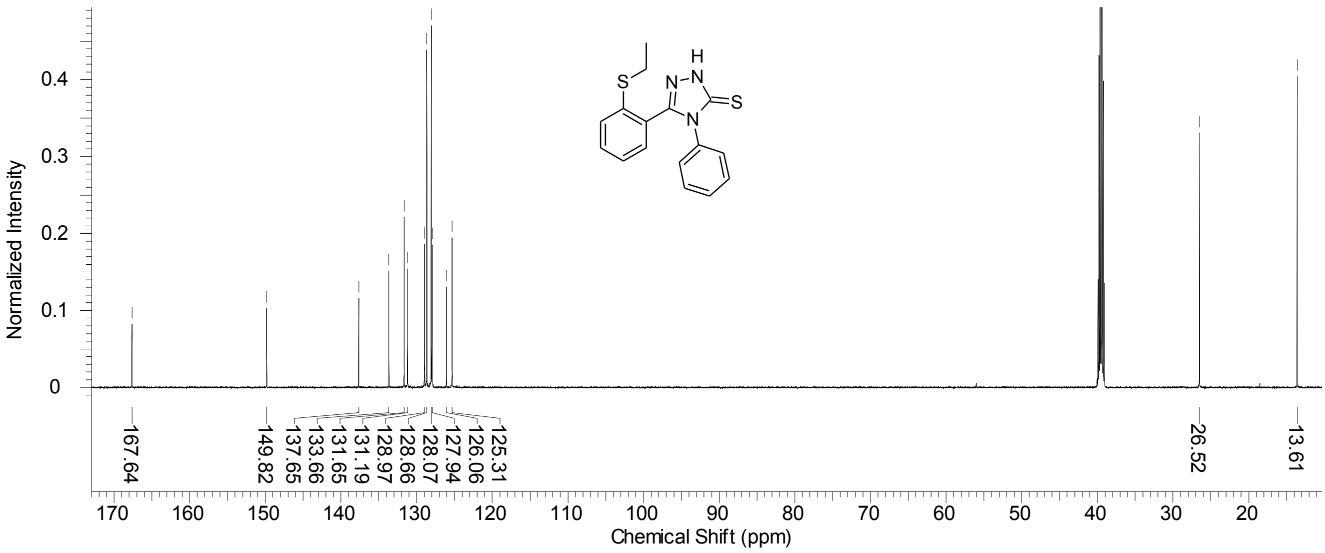

Figure S5. ^{1}H spectrum of compound **3** in DMSO-d_6.

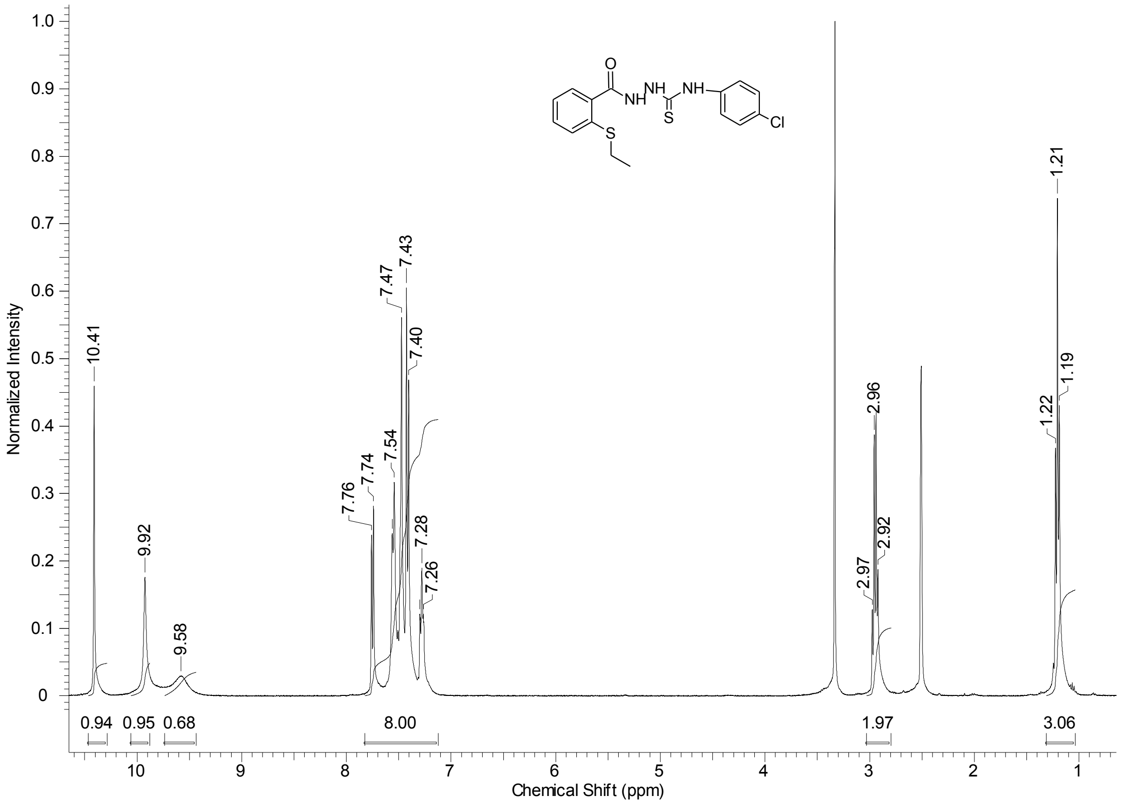

Figure S6. ^{13}C spectrum of compound 3 in DMSO-d_6.

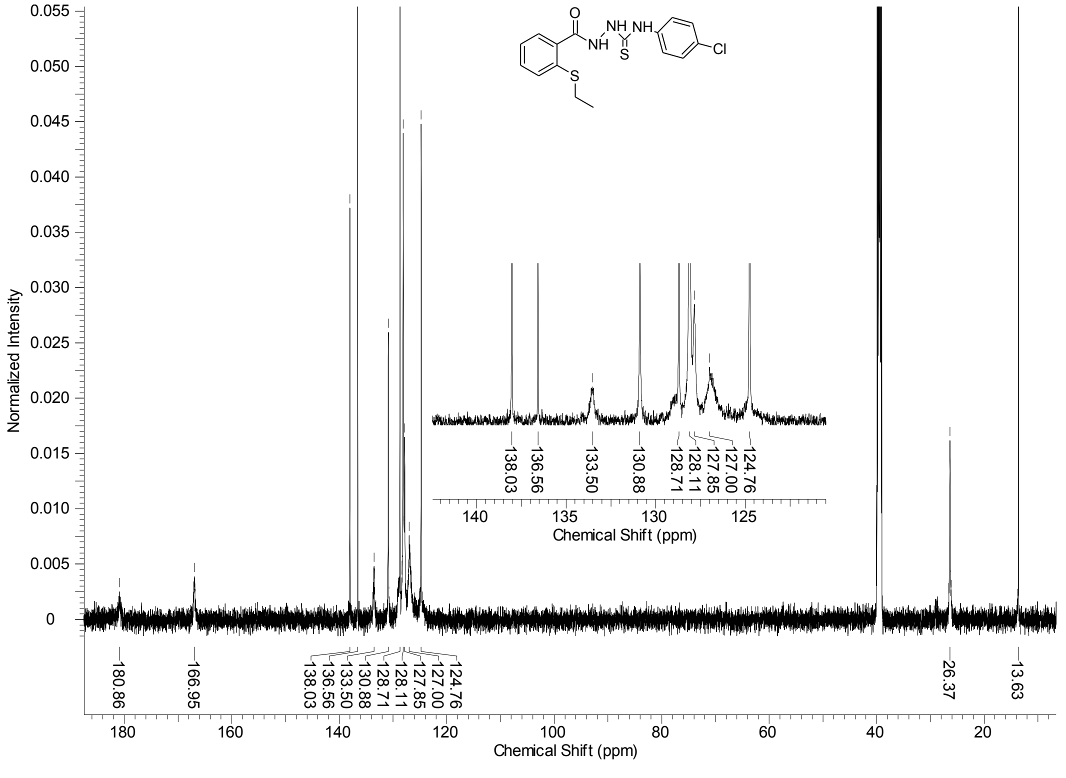

Figure S7. ^{1}H spectrum of compound **8** in DMSO-d_6.

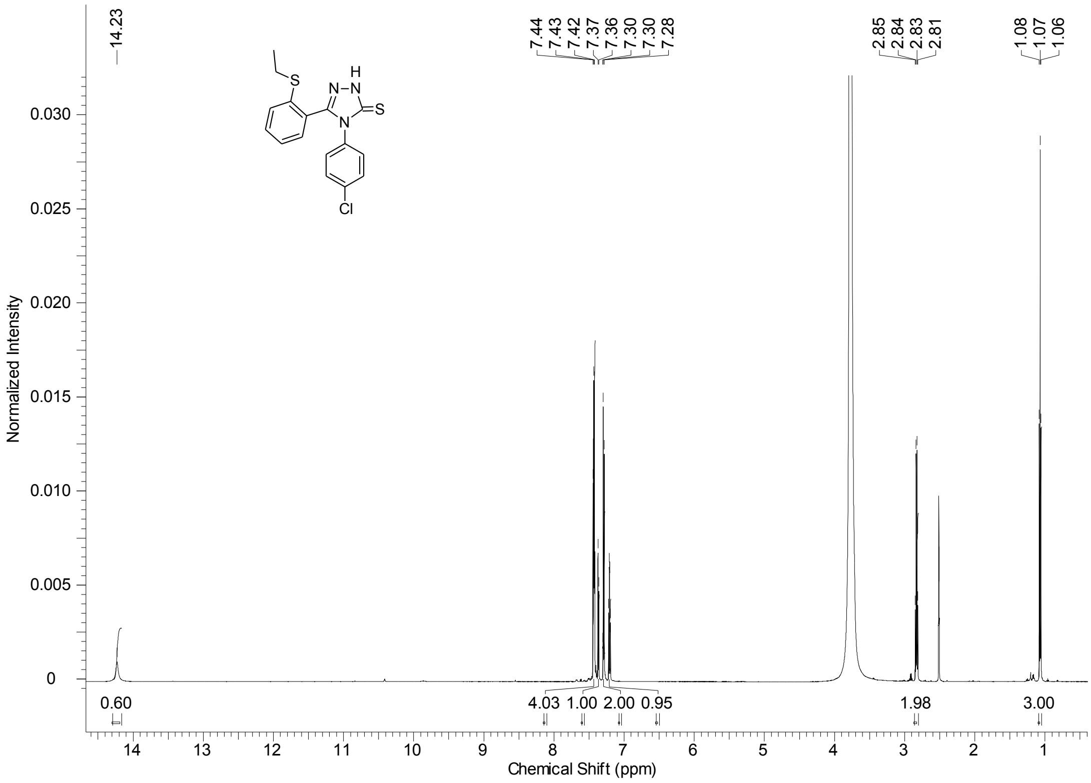

Figure S8. ^{13}C (APT) spectrum of compound **8** in DMSO-d_6.

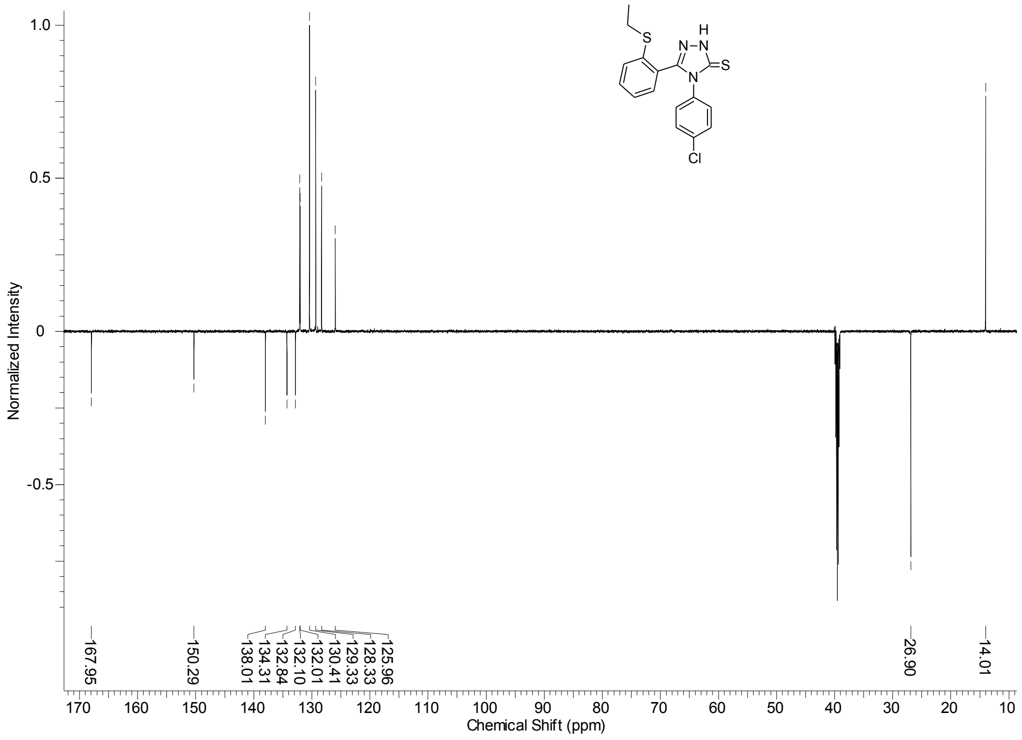

Figure S9. ^{1}H spectrum of compound **4** in DMSO-d_6.

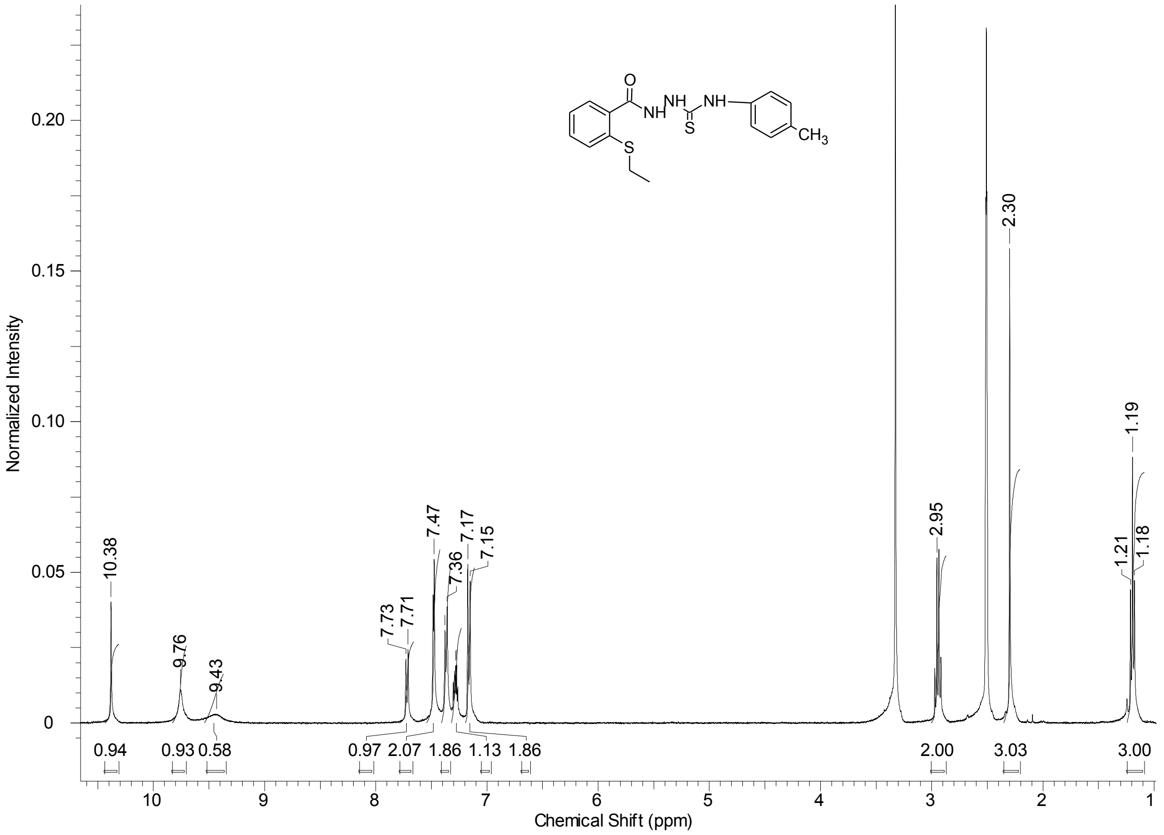

Figure S10. ^{13}C spectrum of compound **4** in DMSO-d_6.

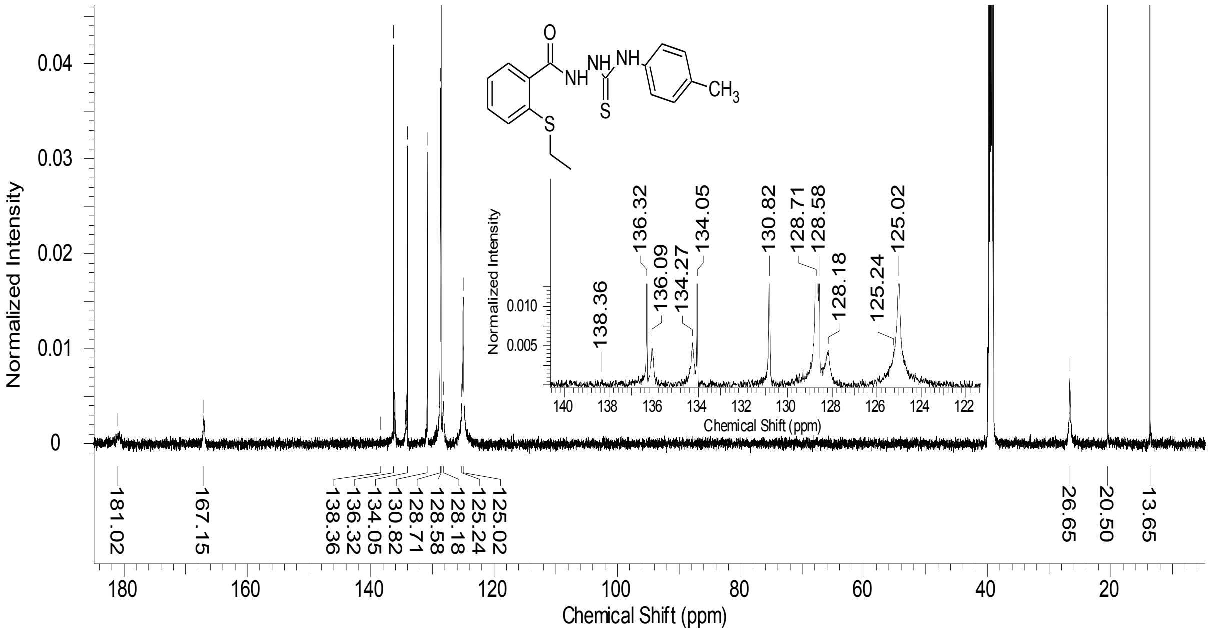

Figure S11. ^{1}H spectrum of compound **9** in DMSO-d_6.

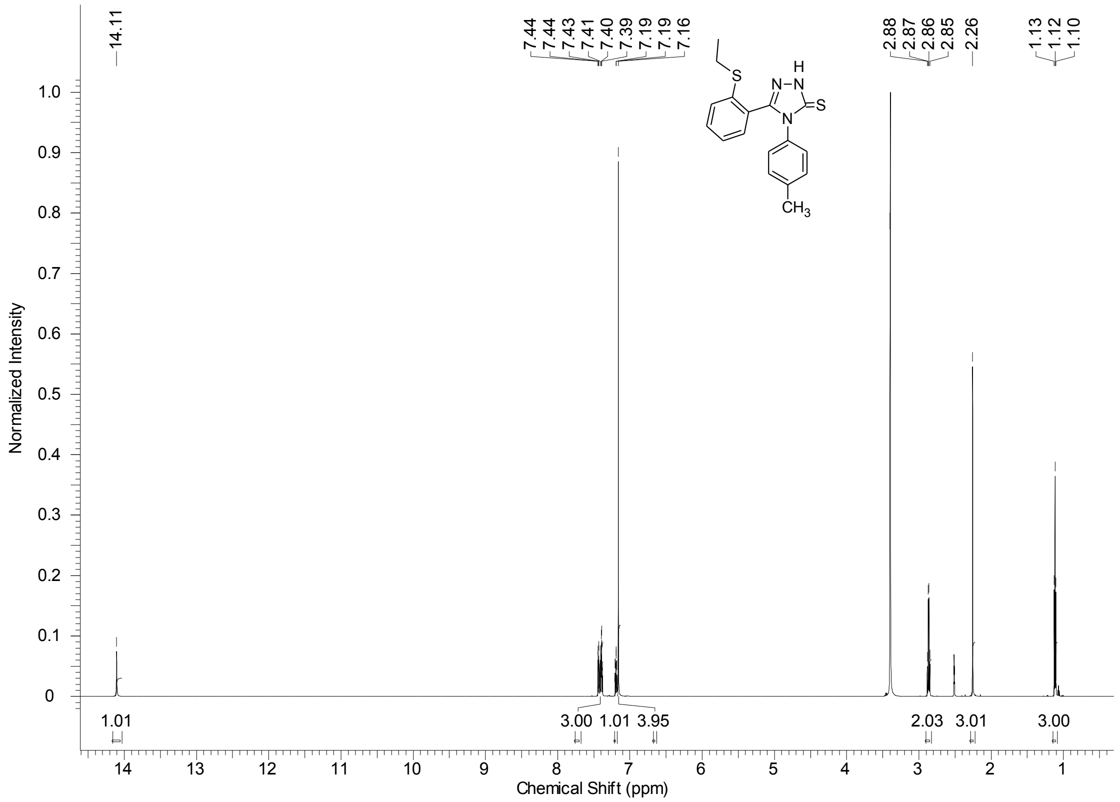

Figure S12. ^{13}C spectrum of compound **9** in DMSO-d_6.

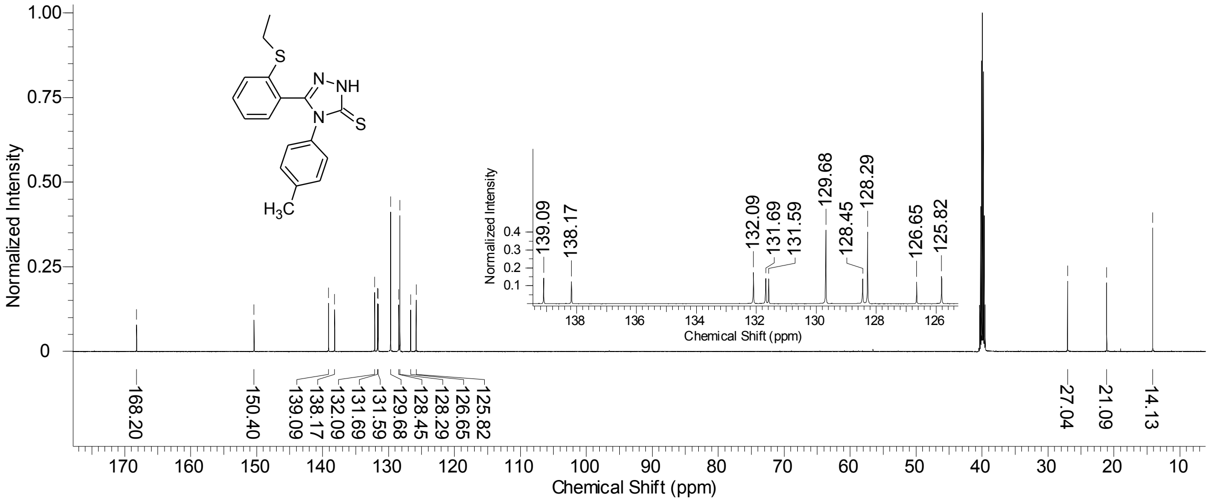

Figure S13. ^{1}H spectrum of compound **5** in DMSO-d_6.

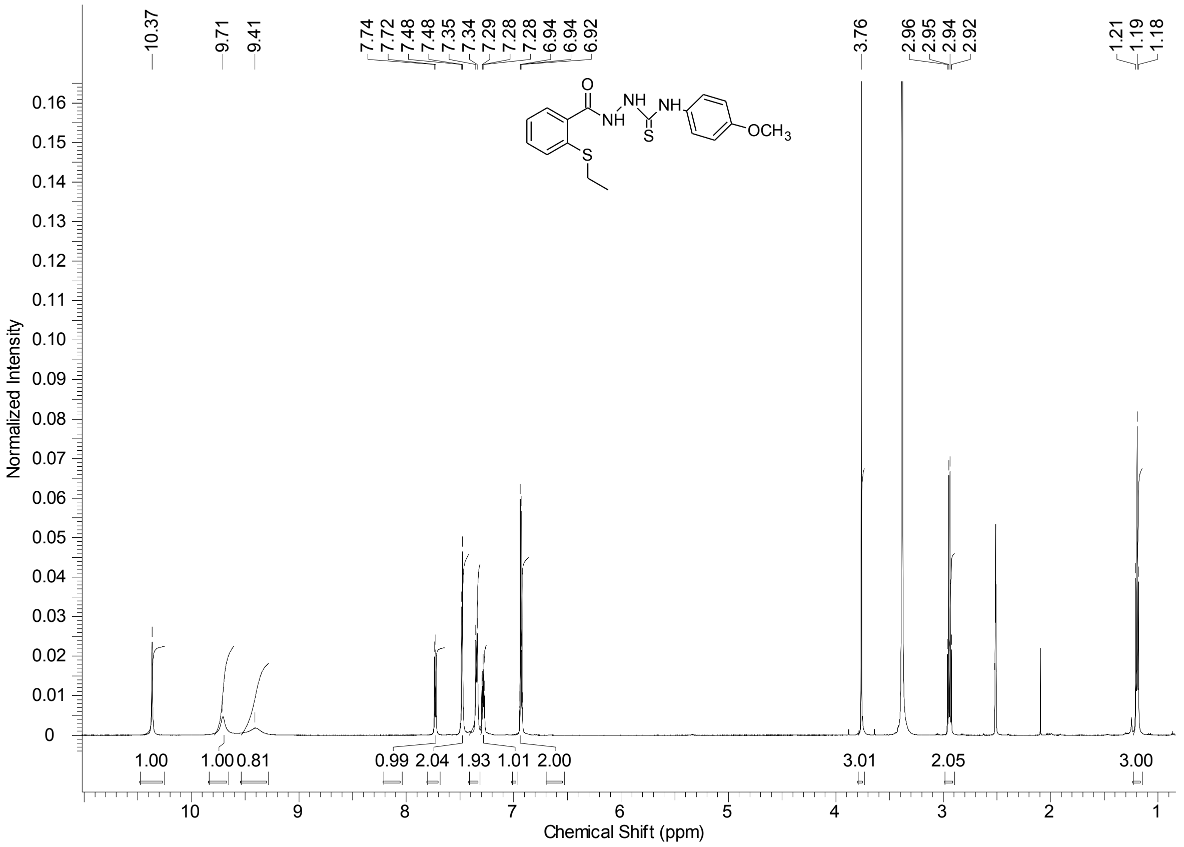

Figure S14. ^{13}C spectrum of compound **5** in DMSO-d_6.

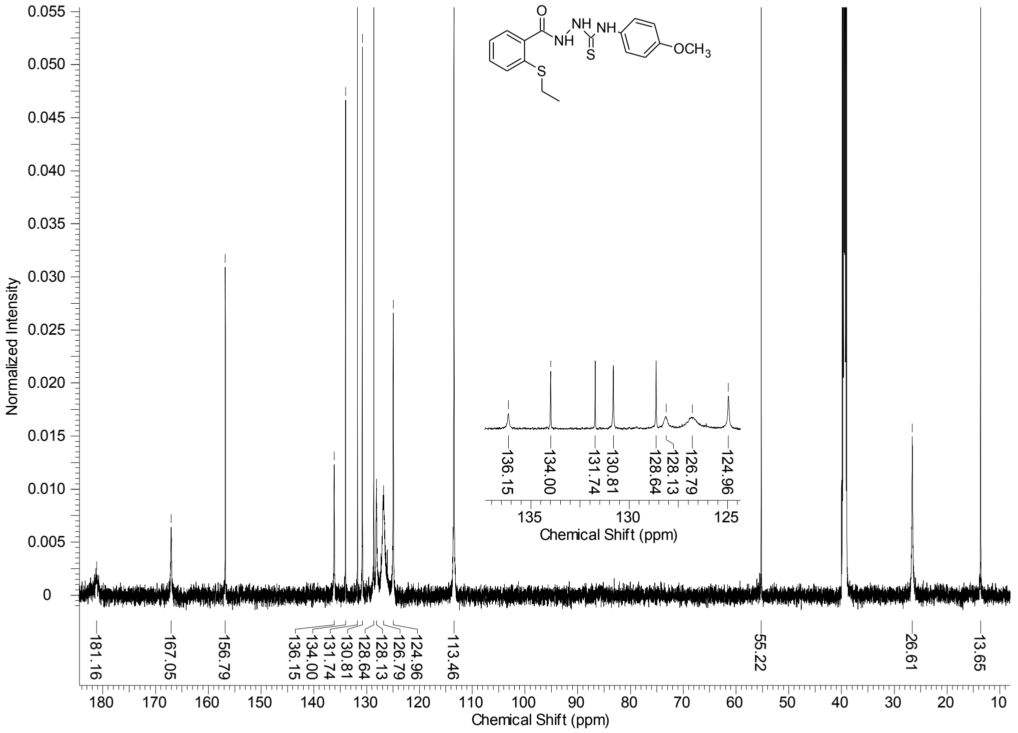

Figure S15. ^{1}H spectrum of compound **10** in DMSO-d_6.

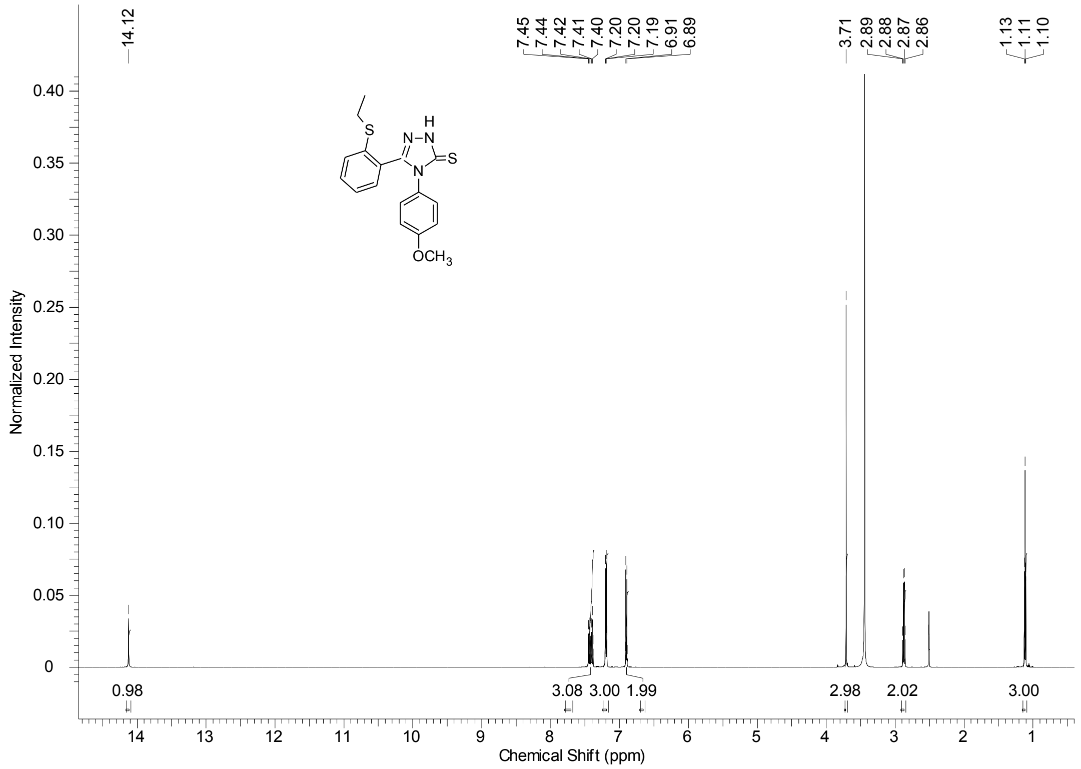

Figure S16. ^{13}C spectrum of compound **10** in DMSO-d_6.

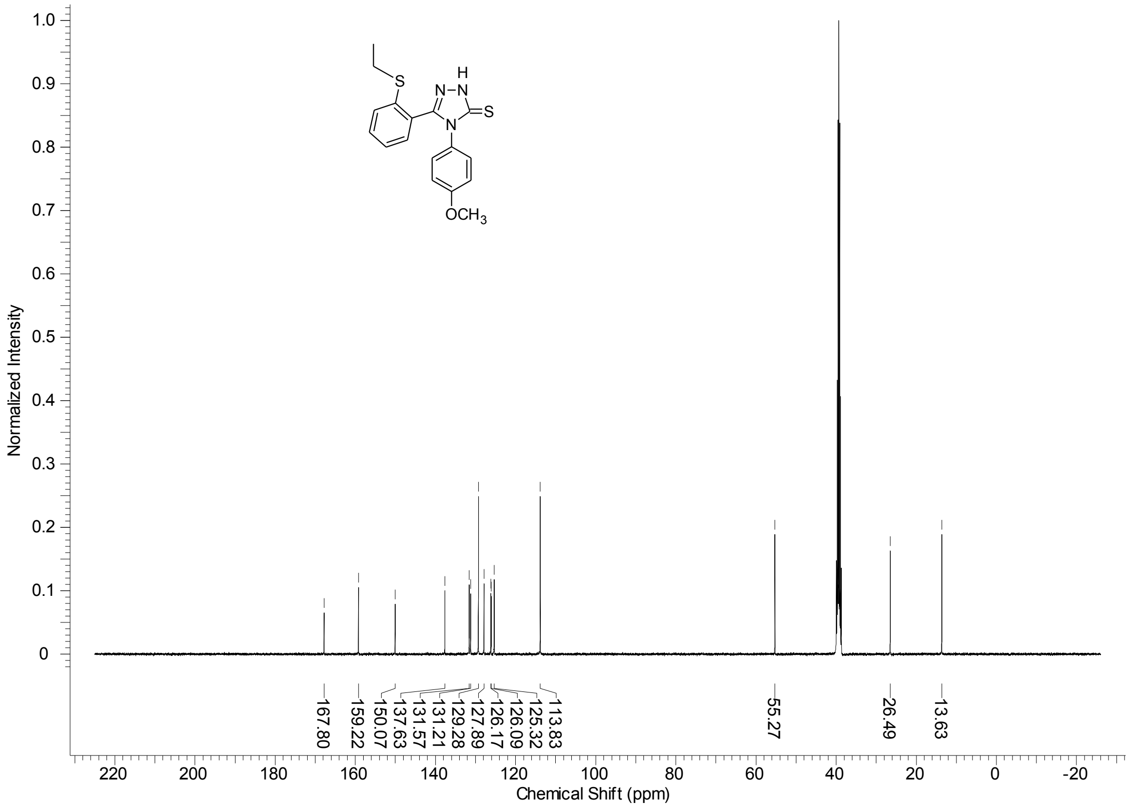

Figure S17. ^{1}H spectrum of compound **6** in DMSO-d_6.

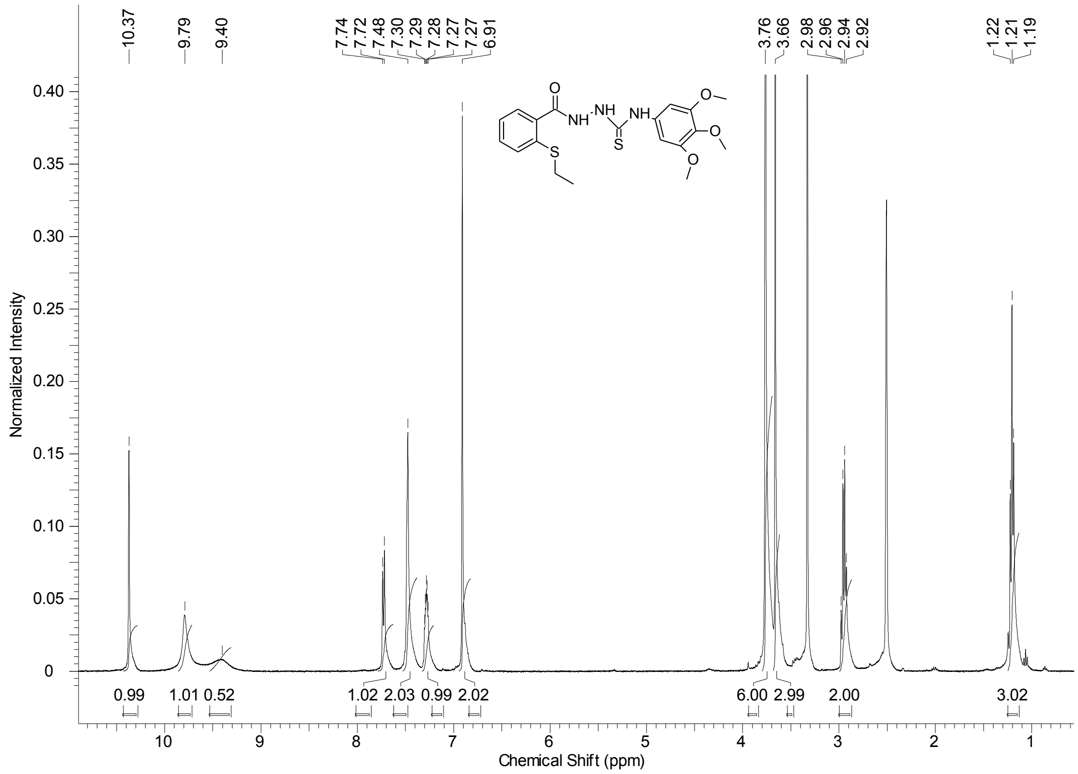

Figure S18. ^{13}C spectrum of compound **6** in DMSO-d_6.

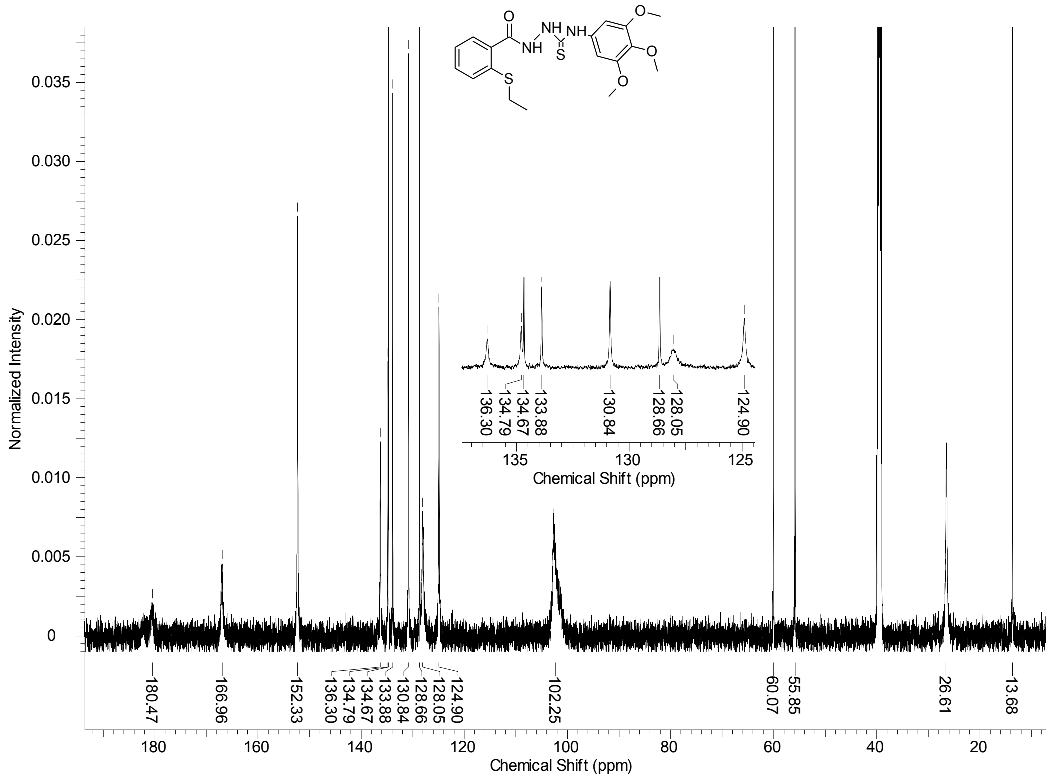

Figure S19. ^{1}H spectrum of compound **11** in DMSO-d_6.

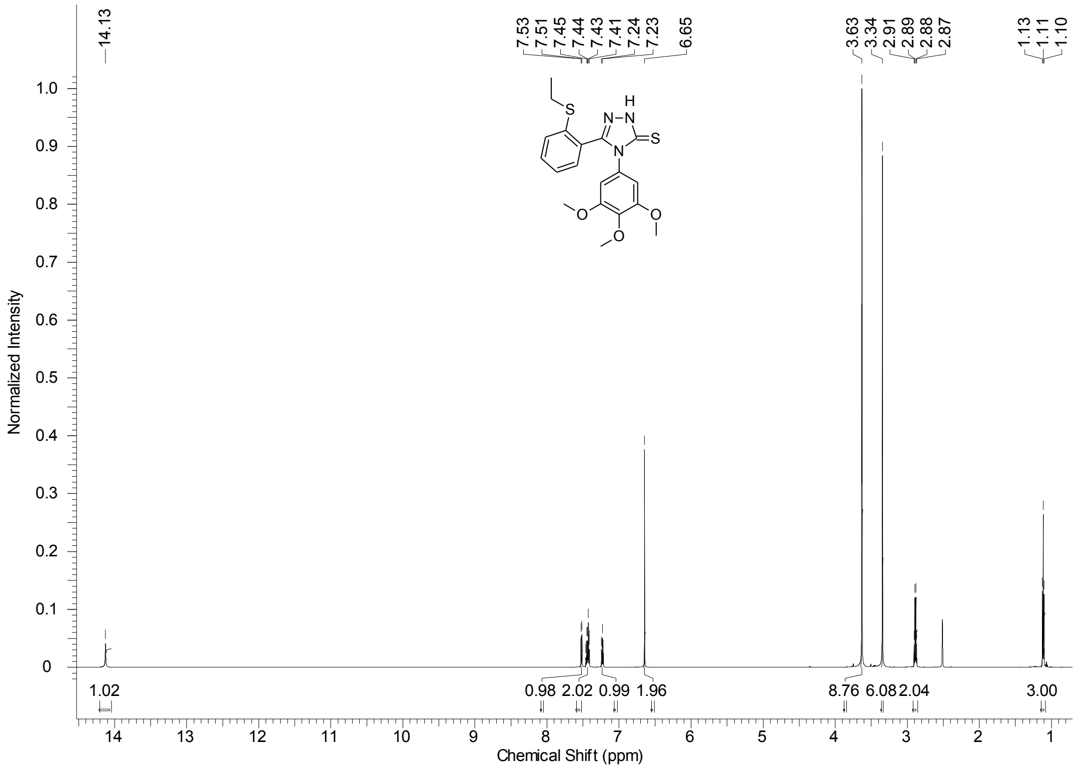

Figure S20. ^{13}C (APT) spectrum of compound **11** in DMSO-d_6.

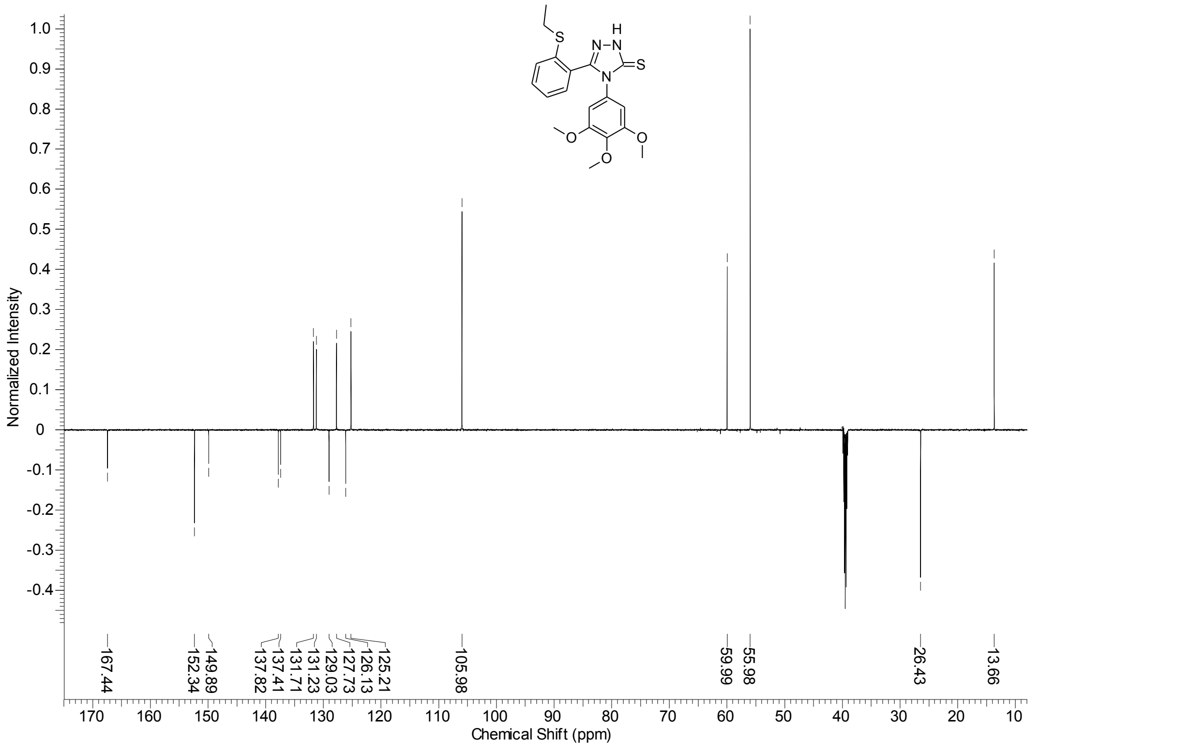

Computational Supporting Data

Compound **3r1** [# opt freq ub3lyp/6-31g(d,p)]

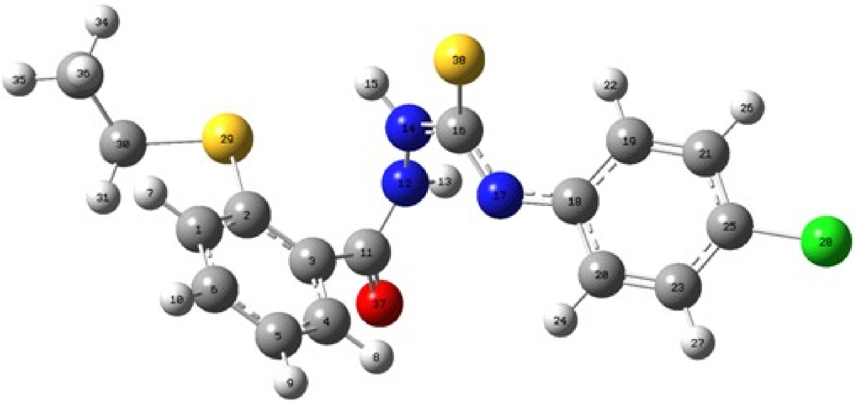

Zero-point correction = 0.284468 (Hartree/Particle)
Thermal correction to Energy = 0.306813
Thermal correction to Enthalpy = 0.307757
Thermal correction to Gibbs Free Energy = 0.227641
Sum of electronic and zero-point Energies = −2114.485647
Sum of electronic and thermal Energies = −2114.463302
Sum of electronic and thermal Enthalpies = −2114.462358
Sum of electronic and thermal Free Energies = −2114.542474

	E (Thermal)/KCal/Mol	CV/Cal/Mol-Kelvin	S/Cal/MolKelvin
Total	192.528	82.424	168.619

Mulliken atomic spin densities:

		1
1	C	0.009937
2	C	−0.119027
3	C	−0.007243
4	C	−0.006443
5	C	−0.000279
6	C	0.015678
7	H	−0.000625
8	H	−0.001201
9	H	0.001046
10	H	−0.000702
11	C	−0.040618
12	N	0.113716
13	H	0.008464
14	N	−0.037928
15	H	−0.231644

16	C	0.114501
17	N	−0.017897
18	C	0.003119
19	C	−0.028430
20	C	0.017892
21	C	−0.003497
22	H	−0.000117
23	C	0.009474
24	H	−0.002443
25	C	−0.000251
26	H	−0.000077
27	H	0.000351
28	Cl	0.000027
29	S	0.795159
30	C	0.324939
31	H	−0.002150
32	H	−0.020897
33	C	0.006649
34	H	−0.000076
35	H	0.012745
36	H	0.002234
37	O	0.003964
38	S	0.081649

Sum of Mulliken atomic spin densities = 1.00000

Compound **3r2 (radical)** [# opt freq ub3lyp/6-31g(d,p)]

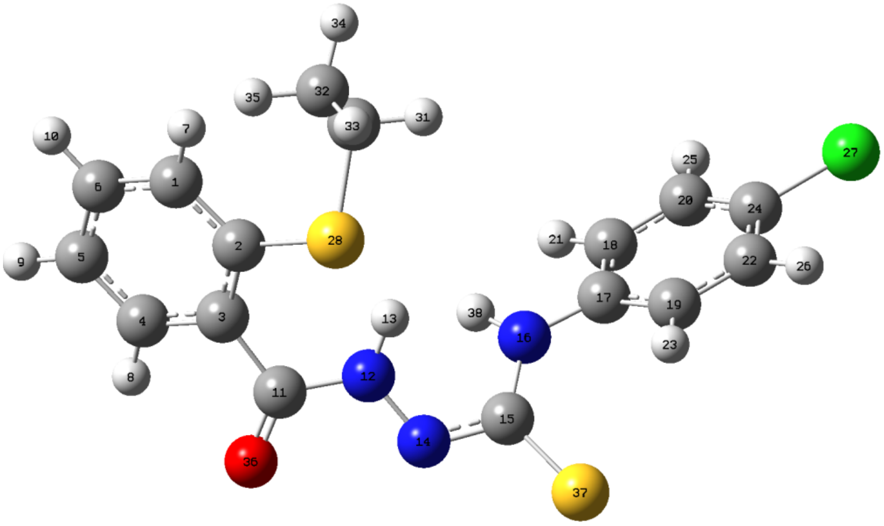

Zero-point correction = 0.284621 (Hartree/Particle)
Thermal correction to Energy = 0.306865
Thermal correction to Enthalpy = 0.307809
Thermal correction to Gibbs Free Energy = 0.227583
Sum of electronic and zero-point Energies = −2114.478768
Sum of electronic and thermal Energies = −2114.456524
Sum of electronic and thermal Enthalpies = −2114.455580
Sum of electronic and thermal Free Energies = −2114.535806

	E (Thermal)/KCal/Mol	CV/Cal/Mol-Kelvin	S/Cal/MolKelvin
Total	192.560	82.380	168.849

Mulliken atomic spin densities:

		1
1	C	−0.003656
2	C	0.006384
3	C	−0.004068
4	C	0.006164
5	C	−0.002993
6	C	0.007323
7	H	0.000151
8	H	−0.000374
9	H	0.000079
10	H	−0.000334
11	C	−0.005013
12	N	0.136679
13	H	−0.005228
14	N	0.483971
15	C	−0.103702
16	N	0.010095
17	C	−0.006008
18	C	0.009772
19	C	0.008446
20	C	−0.004517
21	H	−0.000450
22	C	−0.005689
23	H	−0.000815
24	C	0.010573
25	H	0.000143
26	H	0.000193
27	Cl	0.001282
28	S	0.007054

29	C	0.000593
30	H	0.000023
31	H	0.000080
32	C	0.000295
33	H	−0.000002
34	H	0.000226
35	H	−0.000011
36	O	0.078624
37	S	0.374997
38	H	−0.000239

Sum of Mulliken atomic spin densities = 1.00000

Compound **3r3 (radical)** [# opt freq ub3lyp/6-31g(d,p)]

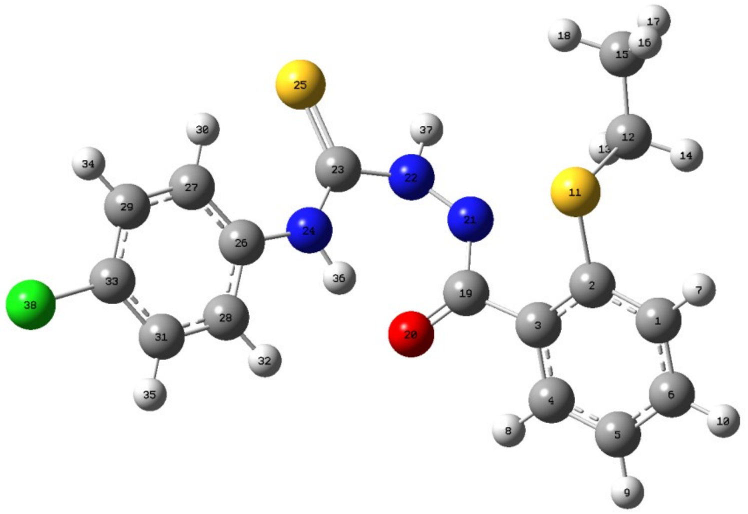

Zero-point correction = 0.286271 (Hartree/Particle)
Thermal correction to Energy = 0.307270
Thermal correction to Enthalpy = 0.307741
Thermal correction to Gibbs Free Energy = 0.230113
Sum of electronic and zero-point Energies = −2114.461291
Sum of electronic and thermal Energies = −2114.439292
Sum of electronic and thermal Enthalpies = −2114.438348
Sum of electronic and thermal Free Energies = −2114.517449

	E (Thermal)/ KCal/Mol	CV/ Cal/Mol-Kelvin	S/ Cal/MolKelvin
Total	193.442	81.329	166.482

Mulliken atomic spin densities:

		1
1	C	−0.001220
2	C	0.000611
3	C	−0.002528
4	C	0.001749
5	C	−0.001823
6	C	0.003473
7	H	−0.000148
8	H	−0.000231
9	H	0.000151
10	H	−0.000177
11	S	0.094268
12	C	−0.003858
13	H	0.002992
14	H	0.000914
15	C	0.000399
16	H	−0.000004
17	H	−0.000092
18	H	−0.000082
19	C	0.005938
20	O	0.149833
21	N	0.397865
22	N	0.237919
23	C	−0.052580
24	N	0.022300
25	S	0.140571
26	C	−0.004957
27	C	0.009213
28	C	0.010456
29	C	−0.006053
30	H	−0.001010
31	C	−0.005108
32	H	−0.000286
33	C	0.011707
34	H	0.000221
35	H	0.000176
36	H	−0.002017
37	H	−0.010171
38	Cl	0.001588

Sum of Mulliken atomic spin densities = 1.00000

<u>Compound **3 (neutral)** [opt freq b3lyp/6-31g(d,p)]</u>

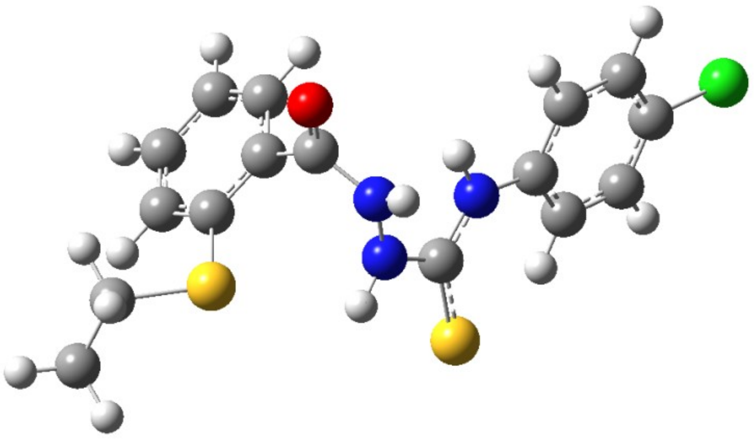

Zero-point correction = 0.296816 (Hartree/Particle)
Thermal correction to Energy = 0.318665
Thermal correction to Enthalpy = 0.319609
Thermal correction to Gibbs Free Energy = 0.241849
Sum of electronic and zero-point Energies = −2115.146198
Sum of electronic and thermal Energies = −2115.124349
Sum of electronic and thermal Enthalpies = −2115.123405
Sum of electronic and thermal Free Energies = −2115.201165

	E (Thermal)/KCal/Mol	CV/Cal/Mol-Kelvin	S/ Cal/MolKelvin
Total	199.965	81.878	163.660

<u>Compound **8 (radical)** [opt freq ub3lyp/6-31g(d,p)]</u>

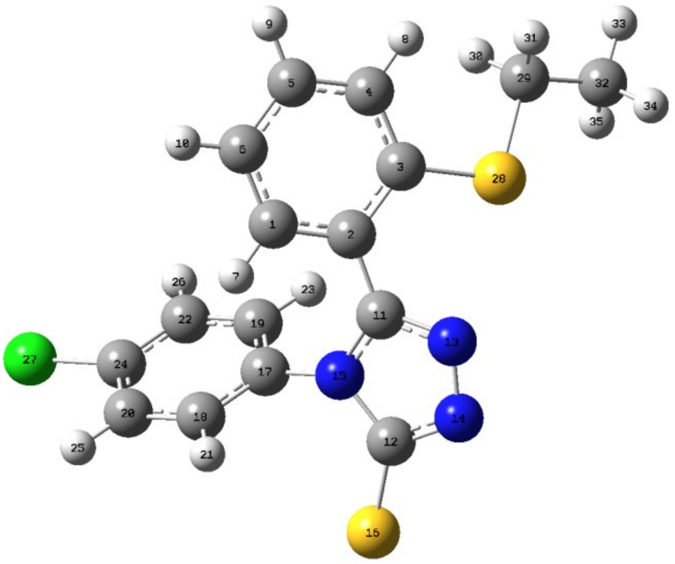

Zero-point correction = 0.257555 (Hartree/Particle)
Thermal correction to Energy = 0.277493
Thermal correction to Enthalpy = 0.278437
Thermal correction to Gibbs Free Energy = 0.205137
Sum of electronic and zero-point Energies = −2038.106883
Sum of electronic and thermal Energies = −2038.086946
Sum of electronic and thermal Enthalpies = −2038.086001
Sum of electronic and thermal Free Energies = −2038.159302

	E (Thermal)/KCal/Mol	CV/ Cal/Mol-Kelvin	S/ Cal/Mol-Kelvin
Total	174.129	74.461	154.273

Mulliken atomic spin densities:

		1
1	C	−0.010589
2	C	0.021197
3	C	−0.023838
4	C	0.022631
5	C	−0.009553
6	C	0.020267
7	H	0.000635
8	H	−0.000716
9	H	0.000582
10	H	−0.000940
11	C	0.161590
12	C	−0.064291
13	N	0.016363
14	N	0.210826
15	N	0.009669
16	S	0.641561
17	S	0.002543
18	C	0.000568
19	H	−0.000055
20	H	0.000126
21	C	0.000021
22	H	−0.000003
23	H	0.000020
24	H	−0.000002
25	C	−0.000820
26	C	0.001642
27	C	0.000569
28	C	−0.000764

29	H	−0.000523
30	C	−0.000084
31	H	−0.000099
32	C	0.001169
33	H	0.000081
34	H	0.000039
35	Cl	0.000179

Sum of Mulliken atomic spin densities = 1.00000

Compound **8 (neutral)** [opt freq b3lyp/6-31g(d,p)]

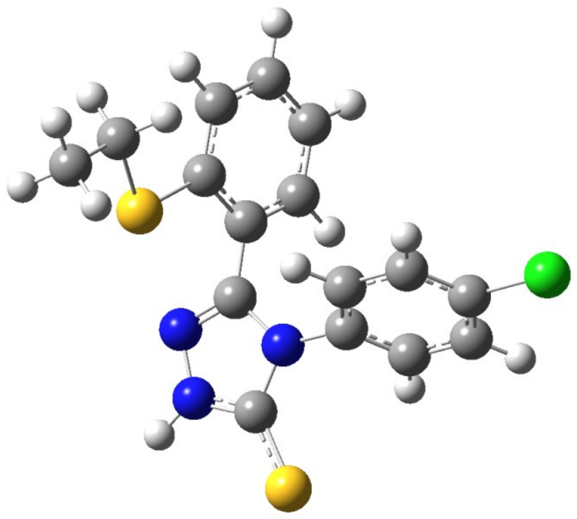

Zero-point correction = 0.270712 (Hartree/Particle)
Thermal correction to Energy = 0.291003
Thermal correction to Enthalpy = 0.291947
Thermal correction to Gibbs Free Energy = 0.217869
Sum of electronic and zero-point Energies = −2038.741688
Sum of electronic and thermal Energies = −2038.721397
Sum of electronic and thermal Enthalpies = −2038.720452
Sum of electronic and thermal Free Energies = −2038.794531

	E (Thermal)/KCal/Mol	CV/ Cal/Mol-Kelvin	S/ Cal/MolKelvin
Total	182.607	75.755	155.911
